# Evolution of group II introns

**DOI:** 10.1186/s13100-015-0037-5

**Published:** 2015-04-01

**Authors:** Steven Zimmerly, Cameron Semper

**Affiliations:** Department of Biological Sciences, University of Calgary, 2500 University Drive N.W., Calgary, Alberta T2N 1N4 Canada

**Keywords:** Ribozyme, Retroelement, Spliceosome, Molecular evolution, Mobile DNA

## Abstract

Present in the genomes of bacteria and eukaryotic organelles, group II introns are an ancient class of ribozymes and retroelements that are believed to have been the ancestors of nuclear pre-mRNA introns. Despite long-standing speculation, there is limited understanding about the actual pathway by which group II introns evolved into eukaryotic introns. In this review, we focus on the evolution of group II introns themselves. We describe the different forms of group II introns known to exist in nature and then address how these forms may have evolved to give rise to spliceosomal introns and other genetic elements. Finally, we summarize the structural and biochemical parallels between group II introns and the spliceosome, including recent data that strongly support their hypothesized evolutionary relationship.

## Review

### Introduction

Investigating the evolution of mobile DNAs involves unique challenges compared to other evolutionary studies. The sequences of mobile DNAs are usually short and evolve rapidly, resulting in limited phylogenetic signals. The elements often transfer horizontally, which prevents the linkage of their evolution to that of their host organisms or other genes in the organism. Finally, many mobile elements themselves consist of multiple components that may have different evolutionary histories. All of these complicating factors apply to group II introns and must be considered when trying to understand their evolutionary history.

Group II intron retroelements consist of an RNA and a protein component. The RNA is a ribozyme (catalytic RNA) that is capable of self-splicing *in vitro*, while the intron-encoded protein (IEP)’s open reading frame (ORF) sequence is contained internally within the RNA sequence and encodes a reverse transcriptase (RT) protein [1-6]. The two components cooperate intricately to carry out a series of inter-related reactions that accomplish intron splicing and retromobility. In addition to the 2- to 3-kb retroelement form, group II introns have evolved into many variant forms and spread throughout all domains of life. They are present in bacteria, archaebacteria, mitochondria, and chloroplasts but are notably excluded from nuclear genomes, with the exception of presumably inert sequences transferred to the nucleus as segments of mitochondrial DNA [7,8].

Group II introns have attracted considerable attention, in part due to their hypothesized relationship to eukaryotic pre-mRNA introns. The purpose of this review is to carefully consider the evidence available regarding the evolutionary history of group II introns. We present a summary of the multiple types of group II introns known to exist in nature and discuss a model for how the variant forms arose and subsequently evolved into spliceosomal introns and other elements.

#### Structure and properties of group II introns

**Figure 1:**
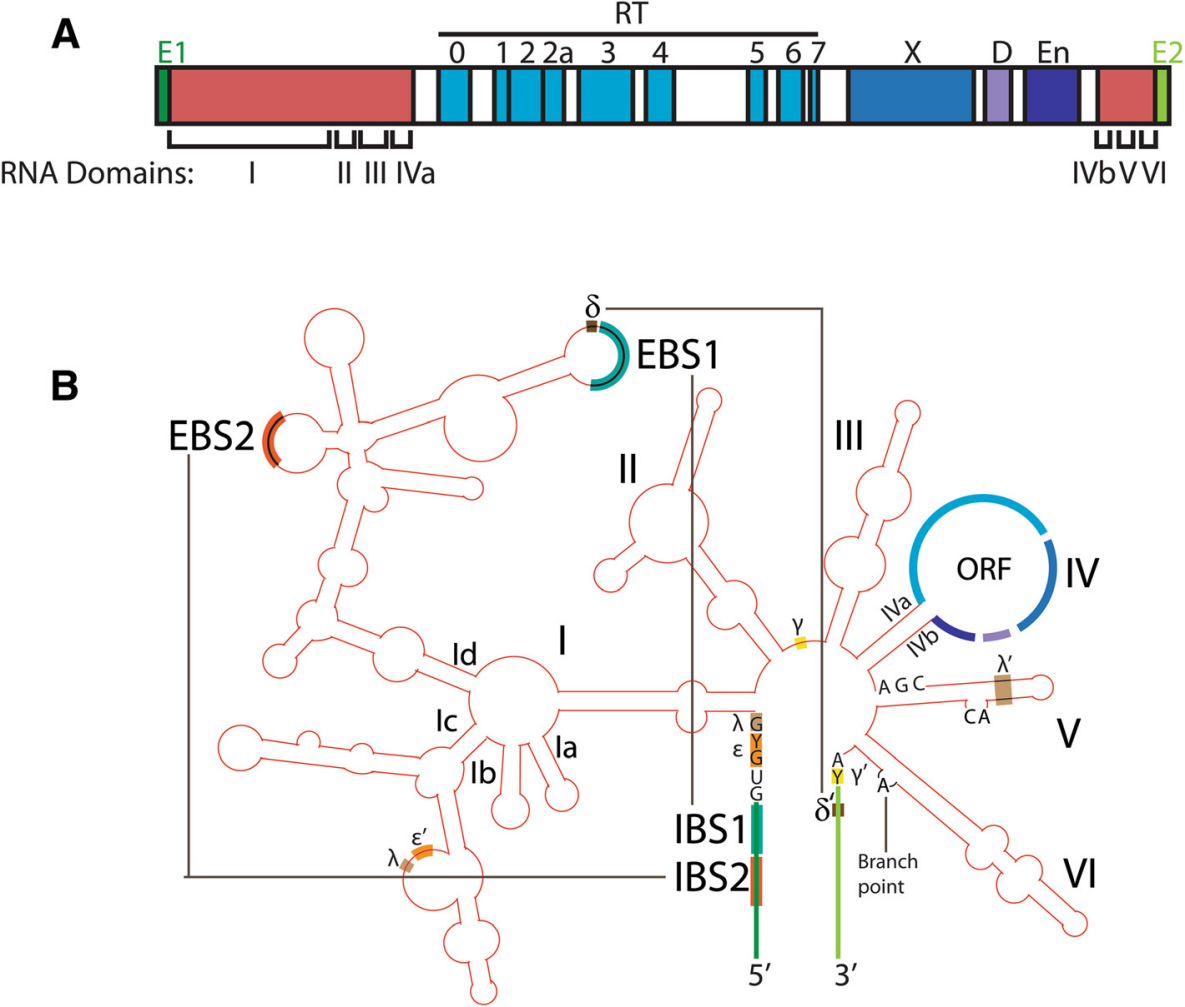
**Group II intron DNA sequence and RNA structure. (A)** Genomic structure of a group II intron. The 2- to 3-kb sequence consists of RNA and protein portions. The intron RNA domains are depicted in red and demarcated with Roman numerals. Domains I to IVa are at the 5′ end of the intron, while domains IVb to VI are at the 3′ end. The IEP sequence is nested within the RNA’s sequence and the domains are denoted by differently shaded blue boxes. The IEP contains a reverse transcriptase domain (RT) with motifs 0 to 7, a maturase domain (X, sometimes called X/thumb), a DNA-binding domain (D), and an endonuclease domain (En). Exons are shown in green. **(B)** Secondary structure of the unspliced RNA transcript. The intron RNA (red) folds into a structure of six domains, with the ORF encoded in a large loop of domain IV. The 5′ and 3′ exons are the green vertical lines at the bottom. Watson-Crick pairing interactions that are important for exon recognition are IBS1-EBS1, IBS2-EBS2, and δ-δ′ (for IIA introns), which are shown with teal, orange, and brown shadings, respectively, and connected with black lines. For IIB and IIC introns, the 3′ exon is recognized instead through an IBS3-EBS3 pairing (not shown). The ε-ε′, λ-λ′, and γ-γ′ interactions are also indicated, because they have potential parallels in the spliceosome (Figure 5); other known tertiary interactions are omitted for simplicity. Both the RNA and DNA structures depicted correspond to the *L. lactis ltrB* intron. EBS, exon-binding site; IBS, intron-binding site; ORF, open reading frame.

**Figure 2:**
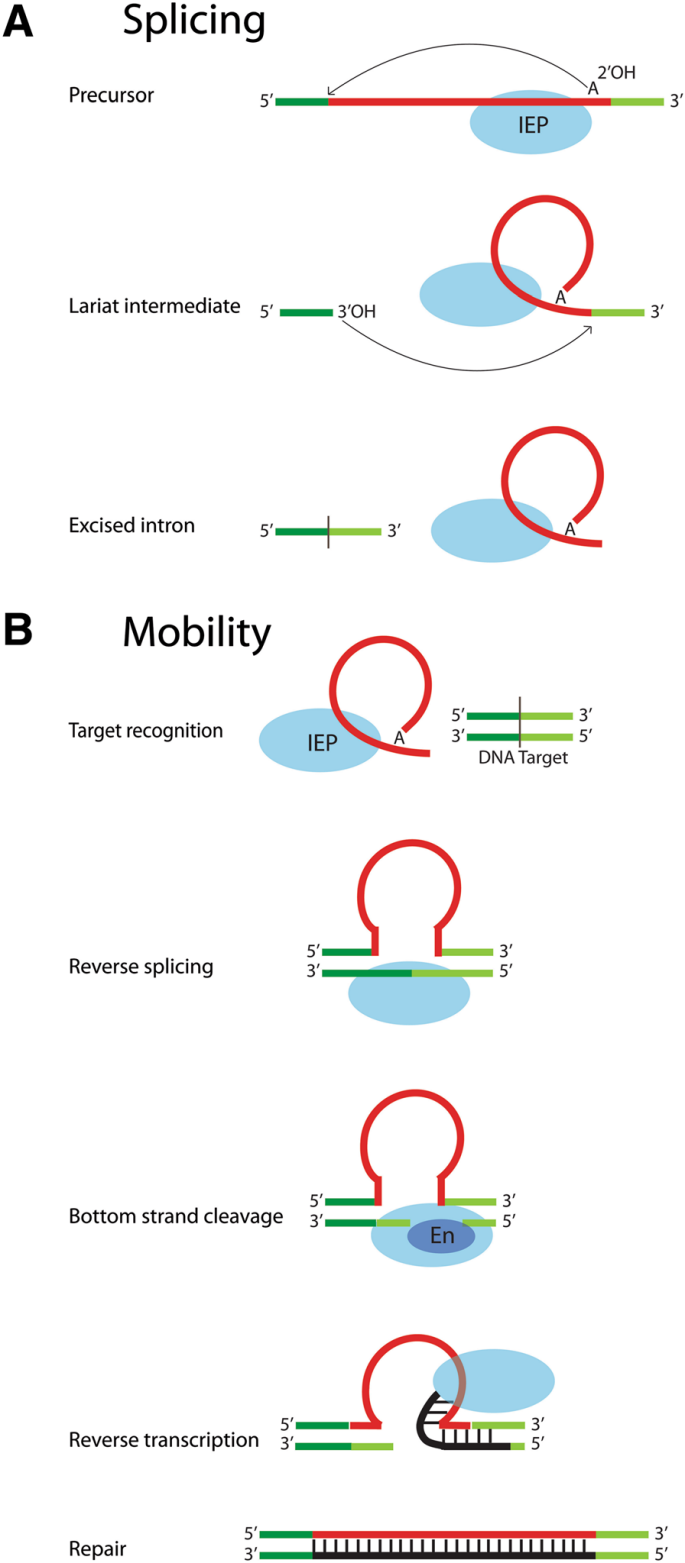
**Group II intron activities. (A)** The splicing reaction. Splicing is intrinsically RNA-catalyzed and occurs for naked RNA *in vitro*; however, under physiological conditions, the IEP is required as well. The IEP binds to the RNA structure to enable it to adopt its catalytic conformation and accomplish splicing. In the first transesterification step of splicing, the 2′OH of the branch site adenosine initiates nucleophilic attack on the 5′ splice junction, yielding cleaved 5′ exon and a lariat-3′ exon intermediate. In the second transesterification, the 3′ OH of the 5′ exon attacks the 3′ splice site to form ligated exons and intron lariat. The IEP remains tightly bound to the lariat to form a mobility-competent RNP particle. **(B)** The mobility reaction, known as target-primed reverse transcription (TPRT). The RNP product of splicing recognizes the DNA target site and reverse splices into the top strand. The En domain cleaves the bottom strand and the free 3′ OH is the primer for reverse-transcription. Host repair activities, which vary across organisms, complete the process. IEP, intron-encoded protein.

The biochemical and genetic properties of group II introns have been described in depth elsewhere [1,3,5,6,9-14] and are summarized briefly here. Of the 2- to 3-kb intron sequence, the RNA component corresponds to approximately 500 to 900 bps, which are separated between the first approximately 600 bp and last approximately 100 bp of the intron sequence (red shading in Figure 1A). After transcription, the RNA folds into a complex structure that carries out splicing [12,14-18]. There is little conservation of primary sequence among all group II intron RNAs, but the introns fold into a common secondary structure that consists of six domains (Figure 1B). Domain I is very large and comprises about half of the ribozyme. Among other roles, it serves as a structural scaffold for the entire ribozyme and importantly recognizes and positions the exon substrates for catalysis [19-21]. Domain V is a small, highly conserved domain that contains the so-called catalytic triad AGC (or CGC for some introns), which binds two catalytically important metal ions [22,23]. Domain VI contains the bulged A motif that is the branch site during the splicing reaction. Splicing is accomplished by two transesterification reactions that produce ligated exons and excised intron lariat (Figure 2A) [24,25]. For some group II introns, the RNA component alone can self-splice *in vitro* under appropriate reaction conditions, typically with elevated concentrations of magnesium and/or salt.

The IEP is encoded within the loop of the RNA domain IV (Figure 1) and is translated from the unspliced precursor transcript. The IEP contains seven sequence blocks that are conserved across different types of RTs, as well as the X domain that is the thumb structure of the RT protein but is not highly conserved in sequence (Figure 1A) [26-29]. Downstream of domain X are DNA binding (D) and endonuclease (En) domains, which are critical for retromobility [30-33].

Both the RNA and IEP are required for splicing and mobility reactions *in vivo*. The translated IEP binds to the unspliced intron structure via the RT and X domains, which results in RNA conformational adjustments leading to splicing (Figure 2A) [34-38]. The role of the IEP in splicing is known as maturase activity because it results in maturation of the mRNA. After splicing, the IEP remains bound to the lariat to form a ribonucleoprotein (RNP) that is the machinery that carries out a retromobility reaction [35,39].

For most group II introns, the mobility reaction is highly specific to a defined target sequence of approximately 20 to 35 bp known as the homing site. The mechanism of mobility is called target-primed reverse transcription (TPRT) [6,10,31,40-44]. The RNP first recognizes and unwinds the two strands of the target, and the intron RNA reverse splices into the top strand of the DNA (Figure 2B). The reaction is the reverse of splicing but utilizes DNA exons rather than RNA exons, and so part of the target site specificity comes from the intron-binding site 1 (IBS1)-exon-binding site 1 (EBS1), IBS2-EBS2, and δ-δ′ pairings between the intron RNA and DNA exons. The IEP facilitates reverse splicing analogously as it does in the forward splicing reaction, that is, it helps the ribozyme fold into its catalytic conformation. In addition, the IEP contributes to target site specificity through interactions of its D domain with the DNA exons. The bottom strand of the target DNA is cleaved by the En domain, either 9 or 10 bp downstream of the insertion site to create a 3′OH that is the primer for reverse transcription of the inserted intron [31,45]. Repair processes convert the inserted sequence to double-stranded DNA, although the repair activities involved differ across host organisms [46-48].

Relevant to this review is a key distinction in the character of group II introns in bacteria compared to introns in mitochondria and chloroplasts. In bacteria, the introns behave mainly as mobile DNAs that survive by constant movement to new genomic sites, whereas in organelles, they are less mobile [5,49,50]. This can be inferred from genome sequences because the majority of intron copies in bacteria are truncated or inactivated, and many are surrounded by other mobile DNAs [49,51]. Most bacterial introns are located outside of housekeeping genes so that their splicing does not greatly affect the host biology. On the other hand, in organelles group II, introns are almost always located in housekeeping genes, which necessitates that they splice efficiently [1,15]. Organellar introns are rarely truncated and frequently have lost mobility properties altogether to become splicing-only entities. As opposed to bacterial introns, organellar introns have taken up a more stable residence in genomes, potentially assuming roles in gene regulation because their splicing factors are under nuclear control (below).

#### Major classes of group II introns

The varieties of group II introns can be classified either according to their RNA or IEP components. Group II introns were initially classified as IIA or IIB based on the RNA sequence and secondary structure characteristics of introns in mitochondrial and chloroplast genomes [15]. A third variation of RNA structure was subsequently identified in bacteria, IIC [52,53]. These three classes each exhibit considerable variation, especially IIB introns, and classes can be further subdivided (for example, IIB1 and IIB2) [15,54]. The most prominent difference among IIA, IIB, and IIC ribozymes is the mechanism of exon recognition, because each class uses a distinct combination of pairing interactions to recognize the 5′ and 3′ exons (that is, different combinations of IBS1-EBS1, IBS2-EBS2, IBS3-EBS3, and δ-δ′ pairings [15,17,19,21,55]).

Alternatively, group II introns can be classified according to phylogenetic analysis of their IEP amino acid sequences. Eight IEP classes have been defined: mitochondrial-like (ML), chloroplast-like (CL), A, B, C, D, E, and F [28,50,56]. The two classification systems are useful for different purposes. Classes IIA, IIB, and IIC apply to all introns regardless of whether they encode an IEP, whereas the IEP-based classes are more specific and correspond to phylogenetic clades. The correspondence between the ribozyme and IEP classifications is shown in Table 1. IIA and IIB introns are found in bacteria, mitochondria, and chloroplasts, while IIC introns are only present in bacteria [15,49,53,57]. Among IEP-classified introns, all forms are found in bacteria, whereas only ML and CL introns are found in mitochondria and chloroplasts (Table 2). There is some relation between IEP classes and host organisms. For example, within bacteria, CL2 introns are almost exclusively found in Cyanobacteria, while class B introns are found exclusively in Firmicutes [50,51].

**Table 1:** **Correspondence between RNA- and IEP-based classes**

**IEP-based classes** ^**a**^	**RNA structure-based classes** ^**b**^		
	**IIA**	**IIB**	**IIC**
A	X		
B		X	
C			X
D		X	
E		X	
F		X	
ML	X		
CL		X	

^a^Classes of introns based on phylogenetic groupings of the IEPs. ^b^Classes of introns based on ribozyme secondary structure characteristics. CL, chloroplast-like; ML, mitochondrial-like.

**Table 2:** **Distribution of intron classes in different organisms and organelles**

	**Eubacteria**	**Archaebacteria**	**Mitochondria**	**Chloroplasts**
RNA-based classes^a^				
IIA	X		X	X
IIB	X	X	X	X
IIC	X			
IEP-based classes^b^				
CL	X	X	X	X
ML	X		X	X
A	X			
B	X			
C	X			
D	X	X		
E	X	X		
F	X			

^a^Classes of introns based on the ribozyme structural characteristics. ^b^Classes of introns based on phylogenetic groupings of the IEPs. CL, chloroplast-like; IEP, intron-encoded protein; ML, mitochondrial-like.

#### Intron variations that deviate from the ‘standard’ retroelement form

Reconstructing the evolution of group II introns requires an accounting of all known intron forms and their distribution. Here, we describe the range of variants that differ from the ‘standard’ retroelement form diagrammed in Figure 1.

##### Introns lacking En domains in the IEP

**Figure 3:**
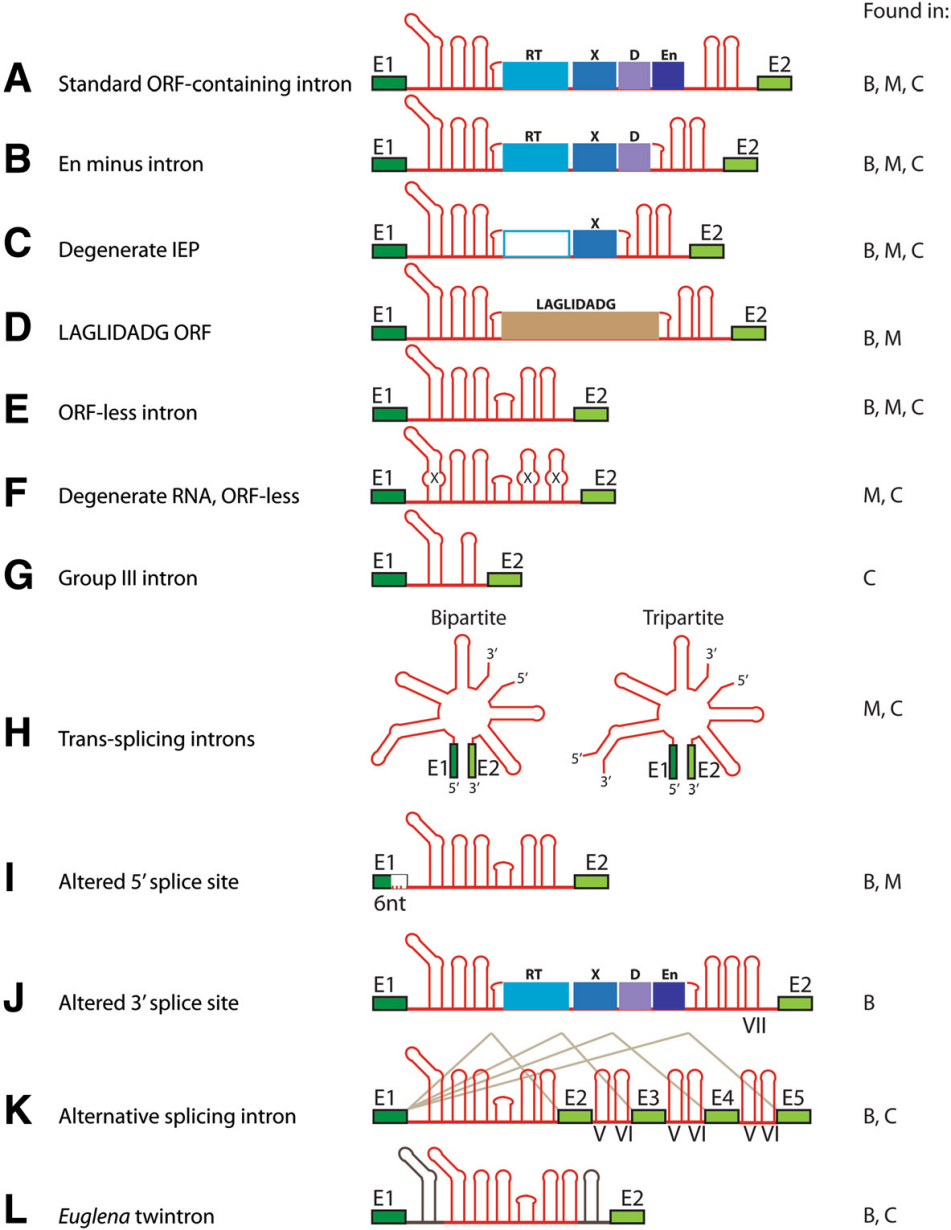
**Variations in group II intron forms.** RNA domains are depicted as stem-loops in red, ORF domains in blue or tan, and exons in green. The right column indicates whether the variants are found in bacteria (B), mitochondria (M), or chloroplasts (C). **(A)** Full-length retroelement form with standard RNA and IEP domains. Example: the IIA intron Ll.LtrB of *Lactococcus lactis*. ORF, open reading frame; RT, reverse transcriptase. **(B)** Intron lacking the endonuclease domain (found in all introns of classes C, D, E, and F and some of class CL). Example: the IIC intron B.h.I1. **(C)** Intron in which the IEP has lost RT motifs while maintaining the domain X/thumb domain required for maturase function. Example: the chloroplast IIA intron *trn*KI1, which encodes the ORF MatK. IEP, intron-encoded protein. **(D)** Intron encoding a LAGLIDADG homing endonuclease. Example: *Grifola frondosa* SSUI1 rRNA intron (fungi). **(E)** ORF-less, self-splicing intron. Example: *S. cerevisiae* aI5g. **(F)** ORF-less intron with a degenerated RNA sequence. Example: tobacco *petD*I1. **(G)** Group III intron. Example: *Euglena gracilis rps*11 **(H)** Trans-splicing group II introns. Examples: tobacco *nad*1I1 (bipartite) and *Chlamydomonas psa*AI1 (tripartite). **(I)** Altered 5′ splice site. Example: *Grifola frondosa* SSUI1 rRNA intron. **(J)** Altered 3′ splice site. Example: *Bacillus cereus* B.c.I4. **(K)** Alternatively splicing group II intron. Example: *Clostridium tetani* C.te.I1. **(L)** Twintron. Example: *Euglena gracilis rps3*.

Approximately a quarter of group II intron IEPs in organelles and over half in bacteria lack an En domain [44,50,51], including all introns of classes C, D, E, and F and a minority of CL introns (Figure 3B). The En domain belongs to the prokaryotic family of H-N-H nucleases [30,58], suggesting that the En domain was appended to an ancestral IEP that had only RT and X domains. If true, then at least some of the lineages of En-minus introns (classes C, D, E, F) represent a form of group II introns that predated acquisition of the En domain.

With regard to mobility mechanisms, En-minus introns are unable to form the bottom strand primer and require an alternative pathway. It has been shown for these introns that the primer is provided by the leading or lagging strand of the replication fork during DNA replication [33,59-62]. Some En-minus introns (namely, IIC/class C) use a different specificity in selecting DNA target sites. Rather than recognizing a homing site of 20 to 35 bp, IIC introns insert at the DNA motifs of intrinsic transcriptional terminators, while a smaller fraction inserts at the *attC* motifs of integrons (imperfect inverted repeat sequences that are recognized by the integron’s integrase) [49,52,63-69].

##### Introns with ‘degenerated’ IEPs that have lost RT activity

Among mitochondrial and chloroplast introns, many IEPs have lost critical RT domain residues (for example, the active site motif YADD) or lost alignability altogether to some of the conserved RT motifs (for example, *trnK*I1 in plant chloroplasts, *nad1*I4 in plant mitochondria, and *psbC*I4 in *Euglena* chloroplasts) (Figure 3C) [27,28,70,71]. These divergent IEPs have undoubtedly lost RT activity and presumably have lost mobility function as well, although the splicing (maturase) function likely endures [27].

A well-studied example is the chloroplast IIA intron *trnK*I1, which is located in an essential tRNA^Lys^ gene. The IEP encoded by this intron, MatK, aligns with other RTs only across motifs 5 to 7, with the upstream sequence being unalignable with motifs 0 to 4; however, domain X sequence is clearly conserved, suggesting the maintenance of the maturase function [27,44]. MatK has been shown biochemically to bind to multiple chloroplast IIA introns, supporting the hypothesis that it has evolved a more general maturase activity that facilitates splicing of multiple IIA introns in plant chloroplasts [70,72].

In bacteria, degenerations of the IEP sequences are rare because the great majority of non-truncated intron copies are active retroelement forms. The only known example is O.i.I2 of *Oceanobacillus iheyensis*, which encodes an IEP of the ML class that lacks the YADD and other motifs. The fact that the ORF has not accumulated stop codons suggests that it retains maturase activity, particularly because its exons encode the DNA repair protein RadC [50].

##### Introns with LAGLIDADG ORFs

A small set of group II introns do not encode RT ORFs but instead encode proteins of the family of LAGLIDADG homing endonucleases (LHEs) and are presumably mobile through a distinct pathway that relies on the LHE (Figure 3D). LHEs in group II introns were first identified in several fungi, although an example has since been identified in the giant sulfur bacterium *Thiomargarita namibiensis* [73-76]. LHEs are a well-studied class of mobility proteins associated with group I introns, and they promote mobility by introducing double-stranded DNA breaks at alleles that lack the introns [2]. Consistent with this role, the LAGLIDADG ORFs in group II introns of the fungi *Ustilago* and *Leptographium* were shown biochemically to cleave intronless target sequences [77,78]. However, the IEP of *Leptographium* did not promote splicing of the host intron, as sometimes occurs for some group I intron-encoded LHEs [77,79]. To date, all identified LHE-encoding group II introns in both mitochondria and bacteria belong to the IIB1 subclass and are located in rRNA genes [73,80].

##### Introns without IEPs

Group II introns without IEPs have lost retromobility properties and exist as splicing-only elements (Figure 3E). They are present in both bacteria and organelles but are especially prevalent in mitochondrial and chloroplast genomes [15]. For example, in plant angiosperms, there are approximately 20 ORF-less group II introns in each mitochondrial and chloroplast genome [70,71,81,82]. These plant organellar introns have been inherited vertically for over 100 million years of angiosperm evolution, consistent with their lack of a mobility-promoting IEP. Because the introns are situated in housekeeping genes in each organelle, efficient splicing is enabled by many splicing factors supplied by the host cells (below). In organellar genomes of fungi, protists, and algae, ORF-less group II introns are also common but less prevalent than in plants. Many of these introns contain remnants of IEP sequences, pointing to a sporadic and ongoing process of loss of the IEP and retromobility [53,83-86].

In bacteria, ORF-less group II introns are rare. Among the known examples, the ORF-less introns nearly always reside in genomes containing related introns whose IEPs may act in *trans* on the ORF-less introns [50]. Splicing function in *trans* has in fact been demonstrated experimentally for an IEP in a cyanobacterium [87]. The sole known exception to this pattern is the C.te.I1 intron in *Clostridium tetani*, for which no IEP-related gene is present in its sequenced genome. C.te.I1 self-splices robustly *in vitro*, and it was speculated that the intron might not require splicing factors *in vivo* [88,89]. This example lends plausibility to possibility that the ribozyme form of group II introns may exist and evolve in bacteria apart from the retroelement form; however, this would be rare because C.te.I1 is the only example of this type among over 1,500 known copies of group II introns in bacteria [90].

##### Introns with ‘degenerated’ ribozymes

Many group II introns in mitochondria and chloroplasts have defects in conserved ribozyme motifs, such as mispaired DV or DVI helices or large insertions or deletions in catalytically important regions (Figure 3F) [15,44,71,91,92]. For such introns, secondary structure prediction with confidence is difficult or impossible, and these introns have presumably lost the ability to self-splice. Consistent with this inference, no plant mitochondrial or chloroplast group II intron has been reported to self-splice *in vitro*.

For introns with compromised ribozyme structures, splicing relies heavily on host-encoded splicing factors [71,93,94]. The catalogue of host-encoded factors is diverse and organism-specific. In yeast mitochondria, the ATP-dependent helicase MSS116 is a splicing factor for multiple self-splicing group I and group II introns [95]. In plant mitochondria and chloroplasts, an array of nuclear-encoded splicing factors has been identified [71,94,96]. Splicing in chloroplasts involves at least 16 proteins that contain motifs of five families of RNA-binding motifs (CRM, PPR, APO, PORR, and TERF families). Some splicing factors (for example, CRS1) are specific to a single chloroplast intron (*atpF*I1), whereas others (for example, CFM2, MatK) aid in splicing multiple introns, which are usually structurally related [97-100]. The situation is similar in mitochondria, where 11 proteins have been identified [71,101]. Additionally, there are four nuclear-encoded, IEP-derived maturases (nMat-1a, nMat-1b, nMat-2a, nMat-2b) that are imported into organelles and are involved in splicing of multiple mitochondrial and possibly chloroplast introns [71,102-105].

These examples illustrate that group II introns have repeatedly lost their splicing capability in organelles. To compensate, cellular splicing factors have evolved independently in different organisms to enable efficient splicing of the introns that lie in housekeeping genes. Similar to the case of ORF-less group II introns, there has been a conversion from retromobility to splicing-only function, and splicing is under the control of the host nuclear genome.

##### Group III introns

The most extreme examples of degenerated RNA structures are group III introns, found in *Euglena gracilis* chloroplasts (Figure 3G) [106]. These introns are approximately 90 to 120 nt in length and sometimes contain only DI and DVI motifs. *Euglena* chloroplasts are replete with >150 group III and degenerated group II introns, many located in essential genes. Because group III introns lack a DV structure, it is thought that a generalized machinery consisting of *trans*-acting RNAs and/or proteins facilitate their excision from cellular mRNAs.

##### Trans-splicing introns

Some group II intron sequences in plant mitochondria and chloroplasts have been split through genomic rearrangements into two or more pieces that are encoded in distant segments of the genome (Figure 3H) [71,107,108]. The intron pieces are transcribed separately and then associate physically to form a tertiary structure that resembles a typical group II intron. The majority of *trans*-splicing introns are split into two pieces with the break point located in DIV. However, the *Oenethera nad5*I3 and *Chlamydomonas psaA*I1 are tripartite, containing breaks in both DI and DIV [108,109]. These and other *trans*-splicing introns require multiple splicing factors for efficient processing. In the case of *psaA*I1 in *Chlamydomonas reinhardtii* chloroplasts, as many as twelve proteins are required in the *trans*-splicing reaction [110,111]. For some introns, the evolutionary timing of the genomic rearrangement can be specified. The *nad1*I1 intron is *cis*-splicing in horsetail, but *trans*-splicing in fern and angiosperms, indicating that the genomic rearrangement occurred after horsetail split from the fern/angiosperm lineage over 250 million years ago [112,113]. No *trans*-splicing introns have yet been reported in bacteria.

##### Altered 5′ and 3′ splice sites

While the vast majority of group II introns splice at specific junction sequences at the boundaries of the introns (5′GUGYG…AY3′), a number of group II introns have attained plasticity that allows them to splice at other points (Figure 3I). A set of fungal rRNA introns was identified that splice 1 to 33 nt upstream of the GUGYG motif. The alteration in splicing property was attributed to specific ribozyme structural changes, including an altered IBS1-EBS1 pairing, and loss of the EBS2 and branch site motifs [74]. These changes were inferred to have evolved independently multiple times. All of the introns are of the IIB1 subclass and the majority encodes a LAGLIDADG IEP [74]. Interestingly, a similar situation was found for the bacterial intron C.te.I1 of *C. tetani*, which exhibits analogous structural deviations and splices eight nucleotides upstream of the GUGYG motif [89]. Alterations of the 3′ splice site have also been reported. About a dozen class B introns are known that contain insertions at the 3′ end of the intron, called domain VII, which result in a shift of splicing to approximately 50 to 70 nt downstream of the canonical 3′AY boundary sequence at the end of domain VI (Figure 3J) [114-116].

##### Alternative splicing

The fact that group II introns can utilize 5′ and 3′ splice sites separated from the 5′GUGYG and AY3′ sequences allows for the possibility of alternative splicing. The first report of this was in *Euglena* chloroplasts, where several group III introns spliced *in vivo* using noncognate 5′ or 3′ splice sites [117,118]. The frequencies of these splicing events, however, were low, being detected by RT-PCR, and the resultant proteins were truncated due to frame shifts and stop codons, which together raise the possibility that this is a natural error rate in splicing rather than regulated alternative splicing *per se*.

In bacteria, alternative splicing at the 3′ splice site was found for B.a.I2 of *Bacillus anthracis*. In that case, two *in vivo*-utilized sites are located 4 nt apart (each specified by a γ-γ′ and IBS3-EBS3 pairing), which result in two protein products, one consisting of the upstream exon ORF alone and the other a fusion of upstream and downstream ORFs [119]. In a more dramatic example, the *C. tetani* intron C.te.I1 utilizes four 3′ splice sites, each specified by a different DV/VI repeat. Each resulting spliced product is a distinct fusion protein between the 5′ exon-encoded ORF and one of four downstream exon-encoded ORFs [88]. The latter example resembles alternative splicing in eukaryotes because several protein isoforms are produced from a single genetic locus (Figure 3K).

##### Twintrons

A twintron is an intron arrangement in which one group II intron is nested inside another intron as a consequence of an intron insertion event (Figure 3L). For a twintron to splice properly, often the inner intron must be spliced out before the outer intron RNA can fold properly and splice [118,120,121]. Twintrons are common in *Euglena* chloroplasts where they were first described, and where approximately 30 of its 160 introns are in twintron arrangements [106]. Several twintrons are known in bacteria; however, splicing of these twintrons does not appear to greatly impact cellular gene expression, because the twintrons are intergenic or outside of housekeeping genes [51,122]. Twintrons in the archaebacterium *Methanosarcina acetivorans* have a particularly complex arrangement [123]. There are up to five introns in a nested configuration but no coding ORFs in the flanking exons. Based on the boundary sequences of the introns, it can be concluded that the introns have undergone repeated cycles of site-specific homing into the sequences of other group II introns. These repeated insertions are balanced by deletions of intron copies through homologous recombination. For these introns, the twintron organizations do not affect host gene expression but provide a perpetual homing site in the genome for group II introns.

#### Molecular phylogenetic evidence for the evolution of group II introns

While there has been much speculation about intron evolution, it remains difficult to obtain direct evidence for specific models. For group II introns, clear phylogenetic conclusions can only be drawn when analyzing closely related introns. This is because only closely related sequences allow the extensive alignments needed for robust phylogenetic signals. Such analyses have indicated multiple cases of horizontal transfers among organisms. Some of the inferred examples are as follows: from an unknown cyanobacterial source to *Euglena* chloroplasts [124]; from unknown sources into a cryptophyte (red alga; *Rhodomonas salina*) [125] or a green alga (*Chlamydomonas*) [126]; between mitochondrial genomes of diatoms and the red alga *Chattonella* [127]; and from the mitochondrion of an unknown yeast to *Kluyveromyces lactis* [127,128]. In bacteria, it was concluded that group II introns from multiple classes have transferred horizontally into *Wolbacchia* endosymbionts, because the resident introns are of different classes [129]. More broadly, horizontal transfers among bacteria appear to be relatively common because many bacteria contain introns of multiple classes [51,130,131].

Beyond identification of horizontal transfers, unfortunately, global phylogenetic analyses result in poor phylogenetic signals because the number of characters available (that is, those that are unambiguously alignable for all introns) decrease to at most approximately 230 aa for the ORF and approximately 140 nt for the RNA [57]. With such reduced-character data sets, clades are clearly identified in bacteria corresponding to classes A, B, C, D, E, F, ML, and CL [28,50,56,132]; however, relationships among the clades are not well supported. Notably, when IEPs of organellar introns are included in trees along with bacterial introns, the organellar IEPs cluster with the ML and CL clades of bacteria, indicating that introns of mitochondrial and chloroplast genomes originated from the ML and CL lineages of bacteria [28]. A global analysis with all known organellar and bacterial intron IEPs is not possible because of extreme sequence divergence of many organellar introns.

The limited phylogenetic resolution for group II introns was attributed to several potential factors [57]. First, the amino acid data sets had substantial levels of saturation (that is, repeated changes per amino acid), which decreased the signal-to-noise ratio. Second, the sequences of some clades had extreme base composition biases that could distort the results (for example, GC-rich genomes have biased amino acid composition that can cause artifacts; this is especially true for class B introns). In addition, there were problematic taxon-sampling effects (differences in trees depending on which intron sequences were included). These complications underscore the difficulty of obtaining rigorous evidence for the evolution of group II introns and the need for exercising caution in drawing interpretations and conclusions. In the future, identifying the basis for these effects may allow for compensation and optimization that may produce more satisfying conclusions.

#### Coevolution of ribozyme and IEP and the retroelement ancestor hypothesis

Over a decade ago, it was noticed that there is a general pattern of coevolution among group II intron IEPs and their RNA structures [53,133]. Specifically, each phylogenetically supported IEP clade corresponds to a distinct RNA secondary structure. Coevolution of RNA and IEP should not be surprising given the intimate biochemical interactions between ribozyme and protein during the splicing and mobility reactions. However, coevolution clearly has not occurred for group I ribozymes and their IEPs. Group I introns have been colonized by four families of IEPs, and there is evidence for a constant cycle of ORF gain and loss from group I ribozymes [134-137].

The principle of coevolution is a central principle to deciphering the history of group II introns. Importantly, it simplifies the reconstruction from two independent histories to a single history. Based on the pattern of coevolution, a model was set forth to explain the history of group II introns, which was called the retroelement ancestor hypothesis [53,133]. The model holds that group II introns diversified into the major extant lineages as retroelements in bacteria, and not as independent ribozymes. Subsequently, the introns migrated to mitochondria and chloroplasts, where many introns became splicing-only elements.

Phylogenetic analyses have in general supported the initial observation of coevolution, because both RNA and IEP trees define the same clades of introns, thereby excluding extensive exchanges between ribozymes and the different classes of IEPs [57]. However, caveats remain. The most obvious one is the fact that some group II introns encode LHE proteins rather than RT proteins. The invasion of group II ribozymes by LHE’s occurred at least once in bacteria and multiple times in fungal mitochondria [74,76]. So far, these exceptions are limited in number and do not significantly undermine the overall pattern of coevolution. A second caveat comes from topology tests between the IEP and RNA trees which indicated a conflict [57] (topology tests are mathematical techniques for evaluating and comparing different trees). As noted in that study, the conflict could be explained by either discordant evolution (reassortment of IEPs and ribozymes) or convergence of RNA or IEP sequences that masks their true evolutionary relationships. While the source of the conflict was not resolved, more recent data support the latter reason (L. Wu, S. Zimmerly, unpublished).

#### A model for the evolution of group II introns

##### Diversification within Eubacteria

**Figure 4:**
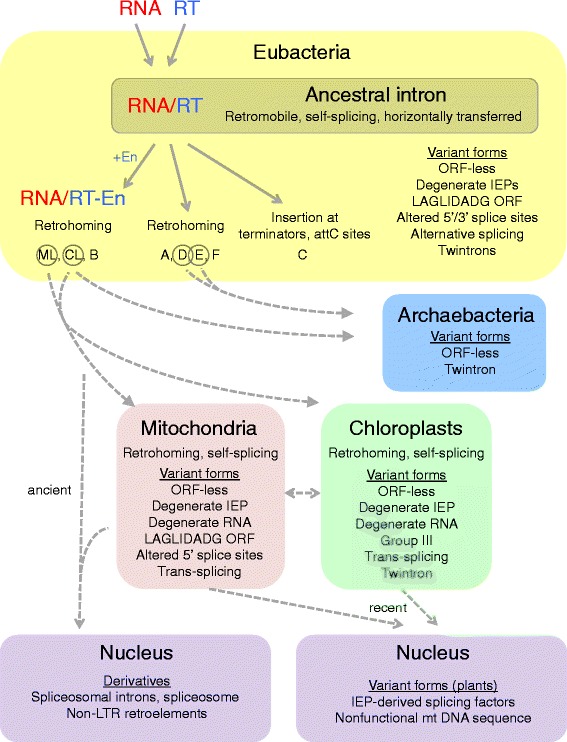
**Global model for group II intron evolution.** An ancient reverse transcriptase combined with a structured RNA to form a group II intron retroelement. This ancestral form was present in Eubacteria and had properties of splicing and retromobility. The retroelement form differentiated into eight lineages, of which ML, CL, and B acquired an endonuclease domain. All lineages but class C (IIC) introns were mobile by retrohoming into site-specific target sequences. Introns of three lineages transferred to archaebacteria, while introns of two lineages transferred to mitochondria and chloroplasts. Variant forms of group II introns were produced in each location as noted. Prior to the LECA, group II introns invaded the nucleus where they developed into the spliceosome and non-LTR retroelements. Much later in plants, group II introns transferred to the nucleus, where the IEPs developed into splicing factors that are imported into mitochondria and/or chloroplasts to help splice organellar group II introns. See text for full description. IEP, intron-encoded protein; LTR, long terminal repeat; ORF, open reading frame; RT,reverse transcriptase.

The retroelement ancestor model continues to be consistent with available data and is elaborated here to show how it can explain the emergence of the known forms and distribution of group II introns (Figure 4). The ancestral group II intron is hypothesized to have been a retroelement in Eubacteria that consisted of a ribozyme and intron-encoded RT component and had both mobility and self-splicing properties. The earliest introns would have behaved as selfish DNAs [49], which then differentiated in Eubacteria into several retroelement lineages (A, B, C, D, E, F, ML, CL). The IEP initially would have consisted of a simple RT, similar to RTs of classes C, D, E, and F, while the En domain was acquired subsequently from H-N-H nucleases present in Eubacteria [30,58]. The En domain would have provided the benefit of enhanced mobility properties and/or allowed the introns to exploit new biological niches.

Of the three target specificities known for bacterial introns (insertion into homing sites, after terminator motifs, and into *attC* sites) [64,65], any of these specificities could have been used by the ancestor, although homing is by far the most prevalent specificity, occurring for all lineages but class C. Horizontal transfers would have driven the dissemination of group II introns across species. Some group II introns took up residence in housekeeping genes, particularly in cyanobacteria and for CL and ML lineages [51,138,139]. These introns would have had to splice efficiently to avoid inhibiting expression of the host genes. Limited numbers of introns deviated from the ‘standard’ retroelement form, including ORF-less introns, introns with degenerate IEPs, twintrons, and alternatively splicing introns. Most of these lost mobility properties but maintained splicing ability. Some introns adapted altered mechanisms of 5′ and 3′ exon recognition and altered 5′ or 3′ intron termini [71,72,74,89,116,117,119,123].

##### Migration to archaebacteria and organelles

Introns belonging to the lineages CL, D, and E migrated from Eubacteria to archaebacteria [51,123]. The direction of migration can be inferred from the lower number and diversity of introns in archaebacteria compared to Eubacteria. Introns of the CL and ML lineages migrated from Eubacteria to mitochondria and chloroplasts. The introns could have been contained within the original bacterial endosymbionts that produced each organelle or been introduced by subsequent migrations. Horizontal transfers of introns among mitochondrial and chloroplast genomes created a diversity of IIA and IIB introns in both organellar genomes [124-128].

##### Diversification within organelles

Within mitochondria and chloroplasts, the character of group II introns changed to become more genomically stable and less selfish. The introns took up residence in housekeeping genes, which necessitated efficient splicing, and which was enabled by host-encoded splicing factors [71,93-96]. While many group II introns maintained retromobility, many more degenerated in their RNA and/or IEP structures or lost the IEPs entirely, leading to immobile introns. In plants, the introns proliferated greatly to copy numbers of approximately 20 per organelle, with nearly all IEPs being lost. At least two IEPs migrated from the plant mitochondrial genome to the nucleus to encode four splicing factors that are imported to the mitochondria and possibly chloroplasts for organellar intron splicing [71,85].

In fungi, a small fraction of ORF-less introns acquired an IEP of the LAGLIDADG family, which permitted mobility through the homing endonuclease mechanism. In mitochondria and chloroplasts, introns sporadically became *trans*-splicing due to genomic rearrangements that split intron sequences [71,107-109,112,113]. In *Euglena* chloroplasts, the introns degenerated on a spectacular scale to become group III introns. The earliest euglenoids are inferred to be intron-poor while the later branching euglenoids harbor more introns, pointing to a process of intron proliferation within *Euglena* chloroplasts [140,141].

##### Caveats

It should be kept in mind that this model is contingent upon the available sequence data. One cautionary note is that our picture of group II introns in bacteria may be skewed, because for the data available the introns were identified bioinformatically in genomes based on the RT ORF. This may result in some oversight of ORF-less group II introns; however, the numbers of those introns do not appear to be large. In a systematic search of bacterial genomes for domain V motifs, nearly all introns identified were retroelement forms [50]. There was one example uncovered of a group II intron with a degenerate IEP, and only a few ORF-less introns, all in genomes with closely related introns where an IEP may act in *trans* on the ORF-less intron. A single independent, ORF-less group II intron was found out of 225 genomes surveyed. Hence, it seems safe to predict that relatively few ORF-less introns have been overlooked in bacteria, unless they have domain V structures unlike those of known group II introns.

#### Origin of group II introns

If the ancestor of extant group II introns was a retroelement, where did that retroelement come from? The simplest scenario is that pre-existing ribozyme and RT components combined into a single element, creating a new mobile DNA. An interesting alternative possibility is that a self-splicing RNA might have arisen at the boundaries of a retroelement to prevent host damage by the mobile DNA [142].

There are many potential sources for the ancestral RT component, because a myriad of uncharacterized RTs exist in bacterial genomes, most of which could potentially correspond to forms that were co-opted by the primordial group II intron [143]. Because there is little evidence that bacterial RTs other than group II introns are proliferative elements, it is possible that the property of mobility emerged only after the RT became associated with the RNA component.

Similarly, there are many structured RNAs in bacteria that could have given rise to the ancestral group II ribozyme, including noncoding RNAs, riboswitches, or even a fragment of the ribosome [144-146]. The primordial RNA component would not necessarily have been self-splicing like modern group II introns, but upon associating with the RT, it would have generated a simple retroelement, which then became specialized and/or optimized to become the efficient retroelement that was then the ancestor of the different lineages. Although the topic of the ultimate origin of group II introns is interesting to consider, any model will be speculative.

Which class of modern group II introns best represents the ancestral group II intron retroelement? It is often claimed in the literature that IIC introns are the most primitive form of group II introns [13,14,18,147]. While this idea is consistent with the small size of IIC introns, it is only weakly supported by phylogenetic data. The study cited provides a posterior probability of only 77% in Bayesian analysis in support of the conclusion (and <50% with neighbor-joining or maximum parsimony methods), whereas 95% is the usual standard for making conclusions with Bayesian analysis [148]. In more recent phylogenetic analyses, IIC introns are also seen often as the earliest branching of group II introns, albeit with weak or inconsistent support [57]. Interestingly, additional classes of group II introns have been uncovered more recently in sequence data, and some of these are as good or better candidates for most ancestral intron (L. Wu, S. Zimmerly, unpublished).

#### Structural parallels between group II introns, spliceosomal introns and the spliceosome

##### Major parallels

The concept that group II introns were the ancestors of spliceosomal introns emerged shortly after the discovery of multiple intron types (spliceosomal, group I, group II introns) [149-151]. Since then, mechanistic and structural evidence has accumulated to the point that few if any skeptics remain. This is a shift from the early years when it was argued that mechanistic constraints could have resulted in convergent evolution of mechanisms and features [152].

The major similarities and parallels for the two intron types are summarized here. In terms of splicing mechanisms, the overall pathways for group II and spliceosomal introns are identical, with two transesterifications and a lariat intermediate (Figure 2A). The chemistry of the two splicing steps share characteristics with regard to their sensitivities to Rp and Sp thiosubstitutions. A Rp thiosubstitution (that is, sulfur atom substituted for the Rp non-bridging oxygen) at the reacting phosphate group inhibits both steps of the reaction for both group II and spliceosomal introns, whereas Sp substitutions do not, suggesting that different active sites are used for the two reactions [153-156]. This contrasts with data for group I introns, for which Rp substitutions inhibited only the first splicing step, and Sp substitutions inhibited only the second step, which is consistent with reversal of a reaction step at a common active site [157,158]. The shared sensitivities for the reactions of group II and spliceosomal introns suggest that similar active sites are used for the two types of introns, with the group II-like active site being maintained during evolution of spliceosomal introns.

**Figure 5:**
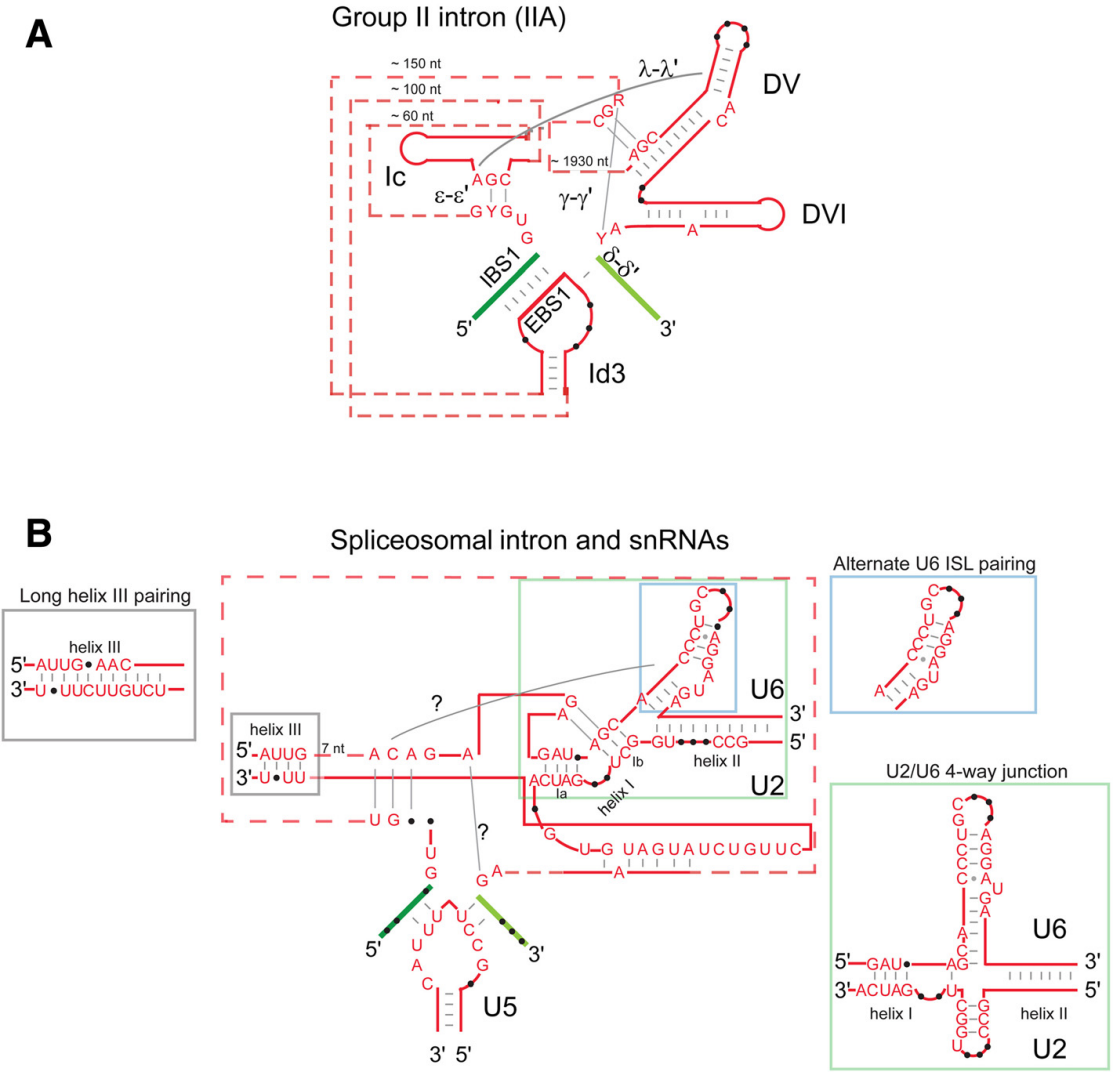
**Structural comparison of group II introns, spliceosomal introns, and snRNAs. (A)** Group IIA intron. EBS, exon-binding site; DV, domain V; DVI, domain VI; IBS, intron-binding site. **(B)** Pairings between U2, U5, and U6 snRNAs and the intron and exons. For both panels, intron sequences and snRNA sequences are shown in red, with exons shown in green. Base pairs are indicated by gray dashes and unpaired nucleotides as black dots. The size of sequences represented by dotted red lines are indicated in nucleotides. For group II introns, selected nucleotide positions critical for splicing are shown, while the sequences shown for snRNAs correspond to the 95% consensus for the U2, U5, and U6 snRNAs sequences present in Rfam [203]. The blue square inset shows an alternative secondary structure model for the ISL of U6, which is less compatible with DV of group II introns but is formed for naked snRNAs. The green square indicates an alternative four-way junction structure, also formed by naked snRNAs. Question marks indicate the interactions found in group II introns for which no equivalent interactions are reported in snRNAs. See text for a full description.

Structurally, there are many parallels between group II intron RNAs and spliceosomal snRNAs, which run the gamut from being clearly analogous to being speculative. The most obvious parallel is the branch site motif that presents the 2′OH of a bulged A to the 5′ splice site for the first step of splicing. For group II introns, the bulged A is contained within a helix of domain VI; in the spliceosome the same bulged structure is formed by the pairing of the U2 snRNA to the intron’s branch point sequence (Figure 5) [159]. Intron boundary sequences are also quite similar and presumably function analogously, being 5′ GU-AY 3′ for group II introns and 5′ GU-AG 3′ for spliceosomal introns (Figure 5). The first and last nucleotides of each intron have been reported to form physical interactions that are essential for an efficient second step of splicing [160-162].

For group II introns, the active site is in domain V, with two catalytically important metal ions being coordinated by the AGC catalytic triad and the AY bulge [147]. A similar structure is formed in the spliceosome by pairings between the U2 and U6 snRNAs, which bear an AGC motif and AU bulge (Figure 5) [23]. The equivalence between the two active sites has been supported experimentally through the substitution of the DV sequence of a group II intron for the analogous positions in the snRNAs of the minor spliceosome (in that case the U12-U6atac snRNA pairing rather than U2-U6) [163]. The substitution demonstrates that the group II intron sequence can assume a functional structure at the putative active site of the spliceosome. More recently, the equivalence of the two active sites was taken to a new level using thiosubstitution and metal rescue experiments, in which a thiosubstitution inhibits a splicing step, but is rescued by metal ions that coordinate sulfur better than magnesium does. These experiments demonstrated that the AGC and bulged AU motifs of the U6-U2 active site coordinate catalytic metal ions as predicted from the crystal structure of the group IIC intron [164].

A further active site parallel comes from the discovery in the group II crystal structure of a triple helix between the AGC base pairs in domain V and two bases of the J2/3 strand (Figure 5A) [147]. This structure is hypothesized to be recapitulated in the active site of the spliceosome, with an AG of the ACAG***A****G*A motif forming the triple base pairs with the ***AG***C of the U6-U2 helix (Figure 5B). Experiments for the yeast spliceosome using covariation-rescue and cross-linking methods support the hypothesized triple base pairs in the spliceosome and lend further support for this active site parallel [165].

A final clear parallel between group II introns and spliceosomal introns was revealed by the crystal structure of a portion of the Prp8 protein, a 280-kDa protein (in yeast) located at the heart of the spliceosome. A region of Prp8 cross-links to the 5′ and 3′ exons and also to the intron’s branch site, indicating its proximity to the spliceosome’s active site. Surprisingly, the crystal structure of a major portion of yeast Prp8 revealed that the cross-linking portion is composed of a reverse transcriptase domain fold [166]. In fact, the existence of an RT domain in Prp8 had been previously predicted correctly based on sensitive sequence pattern profiles [167]. Thus, the active site region of the spliceosome appears to contain remnants of both an ancestral ribozyme (snRNA pairings) and an ancestral group II RT (Prp8), which together strongly support the idea that the eukaryotic spliceosome and nuclear pre-mRNA introns are highly elaborate derivatives of ancient, retromobile group II introns.

##### Less clear yet plausible parallels

Additional parallels between group II intron and spliceosomal intron RNAs are credible but less clear. The loop 1 structure of U5 snRNA is predicted to be analogous the EBS1 loop of group II introns, a substructure that forms base pairs with the 5′ exon of group II introns, thereby delivering the 5′ exon to the active site (Figure 1A). Supporting the parallel, the loop 1 structure of U5 forms cross-links with both the 5′ and 3′ exon boundary sequences [168]. An experiment supporting functional equivalence demonstrated that the EBS1 stem-loop of the bI1 intron of yeast mitochondria could be deleted and then rescued with a stem-loop supplied in *trans* that had either the native bI1 stem-loop sequence or the loop 1 sequence of the U5 snRNA [169]. However, because the function of the EBS1 loop sequence is to form base pairs with the exon’s IBS1, and the U5 loop sequence is fortuitously capable of base pairing with the IBS1 of bI1 (but not other group II introns), the significance of the experiment is less clear. Interestingly, while the EBS1 loop sequence of IIB and IIC introns pairs with only the 5′ exon, the EBS1 loop of IIA introns pairs with both 5′ and 3′ exons (IBS1-EBS1 and δ-δ′ interactions; Figure 1), making the putative parallel more similar for IIA introns than for IIB or IIC introns [170].

The 2-bp ε-ε′ interaction of group II introns has been proposed to be equivalent to an experimentally detected pairing between the U6 snRNA and a sequence near the 5′ end of the intron (Figures 1 and 5) [12,171-173]. While the analogy is reasonable, the U6 pairing was initially reported as 3 bp and later evidence suggested it to be up to 6 bp [174,175]; it remains unclear whether or to what extent the two pairings are analogous structurally and functionally.

Finally, the λ-λ′ interaction of group II introns is a three-way interaction that connects the ε-ε′ interaction (and hence the 5′ end of the intron) to the distal stem of domain V (Figures 1 and 5). The parallel in snRNAs is proposed to be a triple base pair between a subset of nucleotides in the ACAGAGA motif and the internal stem-loop (ISL) helix of U6. While this structural parallel remains a possibility, it appears difficult for the ACAGAGA motif to simultaneously form the ε-ε′-like and λ-λ′-like interactions.

##### Missing or questionable structural parallels

It is important not to ignore features that are not shared between group II and spliceosomal introns, in the rush to pronounce the two types of introns equivalent. Each type of intron has features not found or reported in the other. For example, the γ-γ′ interaction of group II introns is a Watson-Crick base pair between a J2/3 nucleotide and the last position of the intron, but it has not been reported for spliceosomal introns (Figures 1 and 5). The putatively equivalent nucleotides in the snRNAs would be a residue of the ACA***G***AGA box and the last nucleotide (G) of the intron.

Two critical pairings that occur in the spliceosome but not in group II introns are temporal pairings formed during spliceosome assembly but not catalysis [176]. The U1 snRNA pairs to the 5′ end of the intron during splice site recognition and assembly, only to be replaced before catalysis by a pairing between U6 and the 5′ end of the intron. Similarly, the extensive pairings between the U6 and U4 snRNAs occur during spliceosome assembly but are disrupted and replaced by the U6-U2 pairing. Both of these transient RNA-RNA pairings can be predicted to have arisen during the evolutionary advent of the spliceosome, for the purposes of assembly and/or regulation.

On the other hand, Helices Ia and III of the U2-U6 structure (Figure 5) occur during catalysis, but have no equivalent in group II introns, and perhaps even conflict with the structural organization of group II intron RNAs. Helix Ia introduces a spacer between the catalytic AGC motif, the branch site motif and triple helix motif, potentially introducing a structural incompatibility between spliceosomal and group II introns. In any case, group II introns do not have an equivalent helix Ia structure. More problematic is Helix III, which is not present in group II introns, and appears to conflict with proposed structural parallels for the ACAGAGA sequence. In [175], it was proposed that helix III is shortened to approximately 4 bp during catalysis, but might form more fully during assembly. Again, because this established helix has no group II intron equivalent, it may have originated during evolution of the spliceosome.

A modest discrepancy involves the secondary structure of the ISL of U6 and the DV structure of group II introns. The secondary structure of the ISL is usually drawn with an AU bulge opposite an unpaired C (blue square, Figure 5) [177]. However, chemical modification protection data with purified, activated spliceosomes instead suggested an alternative structure that is more similar to group II introns. The alternative structure does not form for naked snRNAs, but it may form in the context of the spliceosome [163,175]. Another perplexing difference between intron types is the break of the catalytic helix into helices 1b and the ISL.

Finally, it is notable that secondary structure models for snRNA pairings have changed over the years, and there are proposed differences in snRNA pairings for yeast versus mammalian snRNAs, despite the fact that the relevant sequences are identical [178-182]. NMR structural analysis of the naked U2-U6 sequences revealed a four-way junction structure (Figure 5B) [180], which was subsequently supported by genetic data in yeast [183]. The four-way junction was proposed to form for the first step, with the three-way junction forming for the second step. However, there is no evidence for the four-way junction structure in the mammalian spliceosome, most recently based on RNA modification protection data of purified, activated U5-U6-U2 spliceosomes [175].

#### The pathway for the evolution of spliceosomal introns from group II introns

Because virtually all eukaryotic genomes contain introns and spliceosomes, with the few exceptions attributed to losses [184-186], the spliceosome was necessarily present in the last eukaryotic common ancestor (LECA). Thus, evolution of ancestral group II introns to the spliceosome would have occurred prior to the LECA. Evidence from genome comparisons indicates that the LECA contained a multitude of introns [187]. Indeed, it is doubtful that such a complex machinery as the spliceosome would have arisen on account of a few introns.

Models for the conversion of group II introns to the spliceosome are not well refined, and multiple scenarios are possible [188-191]. At some point prior to the LECA, group II introns likely invaded the nuclear genome and proliferated as mobile DNAs. The invading group II intron(s) could have come from the genome of the alpha-proteobacterium that became the mitochondrial endosymbiont or alternatively could have been transferred from a bacterium to the nuclear genome after establishment of the mitochondrion. Rampant intron propagation would leave many introns interrupting essential genes, which would require the maintenance of splicing to ensure cell viability. Consequently, the cell evolved splicing factors to facilitate and eventually control splicing of the introns. Debilitating mutations in ribozyme sequences would occur easily through point mutations, leading to many copies of splicing-deficient introns in the genome. On the other hand, discarding such defective introns by precise deletions of entire introns would be rare. The cell could have solved this problem by evolving a general splicing machinery that acts in *trans*, leaving the introns free to lose all their ribozyme structures except for certain boundary sequences. The end result was the transfer of splicing catalysis from individual ribozyme units scattered throughout the genome to a single *trans*-acting RNP machinery that could act on all intron copies.

Because the modern spliceosome is ostensibly a elaborate derivative of a mobile group II intron RNP, it follows that at a time point prior to the LECA, the ribozyme structure of group II introns fragmented into the U2, U5, and U6 snRNA components of the spliceosome. In addition, the RT protein expanded in length through domain accretion, with the fusion of an RNase H domain, MPN/JAB1 (nuclease) domain, and possibly other domains that form portions of the modern 280-kDa Prp8 protein [167,192]. Additional protein splicing factors such as Sm and SR proteins were incorporated into the spliceosomal machinery. The U1 and U4 snRNAs and snRNPs were added as new regulatory or facilitating activities, since they do not have equivalents in group II introns.

One intriguing model for the emergence of the spliceosome predicts that proliferation of mobile group II introns was the driving force for invention of the nuclear membrane [188,193]. The model is based on the likelihood that splicing would have been slow compared to transcription and translation processes. In an uncompartmentalized cell, translation would therefore occur before mRNAs were fully spliced, yielding nonfunctional proteins. By separating transcription and translation, the nuclear membrane ensured that only fully spliced transcripts were translated.

Several studies have experimentally addressed evolutionary issues of group II introns. One series of studies sought to reproduce the fragmentation of a group II ribozyme into a *trans*-splicing intron-in-pieces. It was shown that a retromobile IIA intron could be split into multiple functional *trans*-splicing RNA transcripts, with the break points distributed throughout the sequence and not only in domain IV as occurs for nearly all natural *trans*-splicing introns [189,194,195]. In a separate series of studies, the question was addressed as to why group II introns do not function optimally in nuclear genomes, where they are apparently excluded in functional form in nature. It was found that the introns spliced in the cytoplasm rather than the nucleus and that transcripts were subject to nonsense-mediated decay (NMD) and poor translation. Further dissection showed that transcripts were mislocalized to foci in the cytoplasm and that the excised intron lariat formed RNA-RNA pairings with spliced mRNAs that inhibited their translation. It was inferred that these phenomena demonstrate an incompatibility of group II introns with eukaryotic cellular organization and may have been responsible for the ejection of group II introns from nuclear genomes during evolution [190,196,197].

#### What other elements did group II introns evolve into?

In addition to spliceosomal introns, group II introns are believed to be the ancestors of non-LTR retroelements, a major class of mobile DNAs in eukaryotes [31]. The RTs of group II introns and non-LTR retroelements are related phylogenetically and share sequence motifs 0 and 2a, which are absent from other RTs except diversity-generating retroelements (DGRs) (2a), retroplasmids (2a), and possibly retrons (2a) [143,191,198,199]. Moreover, the retromobility mechanisms of group II and non-LTR elements are similar, with both called target-primed reverse transcription because they involve cleavage of the DNA target to produce a primer for reverse transcription [31,200]. As mobile group II introns were present in the nucleus prior to the LECA, it is plausible that some invading group II introns produced the non-LTR family retroelements in the nucleus through the loss of their ribozyme and splicing functions but retention of mobility functions.

Moreover, it is clear that group II introns spawned other RT-containing units. A subset of CRISPR/Cas elements contain an RT gene, either as a free-standing ORF or fused to a *cas1* gene (denoted G2L1 and G2L2 (group II-like 1 and 2) [143,201]). By sequence, these RTs might be mistaken for group II introns except that no ribozyme RNA structure is present [143]. The *cas1* gene encodes a nuclease that helps integrate short sequences of phage or plasmid into CRISPR arrays, lending cellular immunity to DNAs containing those sequences [202]. The RT genes found within CRISPR/Cas systems are almost certainly derived from group II intron retroelements due to their close sequence similarity. It seems likely that they use a mechanism related to TPRT to integrate the new protospacer sequences into CRISPR arrays.

Three additional types of group II-related RTs exist in bacteria, denoted G2L3, G2L4, and G2L5 [143]. These are not associated with CRISPR/Cas systems and also lack ribozyme structures. It is unknown whether these RTs are part of mobile DNAs or participate in as yet unidentified functions.

## Conclusions

Group II introns are compact and versatile retroelements that have successfully colonized genomes across all domains of life and have given rise to many variant forms. Current data are consistent with the model that the retroelement form (that is, the form diagrammed in Figure 1) was the ancestor of extant group II introns and was the driver for their spread and survival. The evolutionary success of group II introns may be linked to the multifunctionality of their splicing and mobility reactions, which allowed them to spread as selfish DNAs, and then derivatize into adaptable forms that shed either splicing or mobility properties. Interestingly, there is much overlap in variant forms of group II introns found in bacterial and organellar genomes (ORF-less introns, twintrons, altered 5′ splice sites, alternative splicing, degenerate IEP sequences, LAGLIDADG IEPs; Figure 4), which suggests that these derivative forms represent general ways that group II introns can differentiate. The low numbers of derivatives in bacteria suggest that the nonmobile derivatives do not persist long in bacterial genomes, whereas derivatized introns in organelles may persist indefinitely as splicing-only elements, and potentially provide benefits of gene regulation through nuclear control of their splicing.

With regard to the evolutionary pathway of group II introns into spliceosomal introns, important insights over the past 2 years have largely erased doubts about the long-standing hypothesis that the spliceosome descended from group II introns. Indeed, there are no credible competing hypotheses for the origin of the spliceosome. Still, the specifics of the pathway and the full scope of mechanistic parallels remain to be resolved. Additional insight may be forthcoming from structural elucidations of the spliceosome and comparisons to group II intron structures, as well as genomic comparisons of early branching eukaryotes, which may give information about introns in the LECA and potentially suggest evolutionary intermediates or pathways. Overall, the elucidation of group II intron biology, structure, and evolution remains an important facet in understanding the evolution and dynamics of eukaryotic genomes.

## Abbreviations

D: DNA endonuclease domain of the group II intron-encoded protein
DI-DVI: Group II intron domains I-VI
EBS: Exon-binding site
IBS: Intron-binding site
IEP: Intron-encoded protein
LECA: Last eukaryotic common ancestor
LHE: LAGLIDADG homing endonuclease
ORF: Open reading frame
RT: Reverse transcriptase domain of the group II intron-encoded protein
TRPT: Target-primed reverse transcription
X: Maturase domain of the group II intron-encoded protein

## References

[1] Bonen L, Vogel J (2001). The ins and outs of group II introns. Trends Genet.

[2] Belfort M, Derbyshire V, Parker MM, Cousineau B, Lambowitz AM, Craig NL, Craigie R, Gellert M, Lambowitz AM (2002). Mobile introns: pathways and proteins. Mobile DNA II.

[3] Lehmann K, Schmidt U (2003). Group II introns: structure and catalytic versatility of large natural ribozymes. Crit Rev Biochem Mol Biol.

[4] Zimmerly S. Mobile introns and retroelements in bacteria. In: Mullany P, editor. The dynamic bacterial genome. Cambridge University Press; 2005. p. 121-150.

[5] Toro N, Jimenez-Zurdo JI, Garcia-Rodriguez FM (2007). Bacterial group II introns: not just splicing. FEMS Microbiol Rev.

[6] Lambowitz AM, Zimmerly S (2011). Group II introns: mobile ribozymes that invade DNA. Cold Spring Harb Perspect Biol.

[7] Knoop V, Brennicke A (1994). Promiscuous mitochondrial group II intron sequences in plant nuclear genomes. J Mol Evol.

[8] Lin X, Kaul S, Rounsley S, Shea TP, Benito MI, Town CD (1999). Sequence and analysis of chromosome 2 of the plant Arabidopsis thaliana. Nature.

[9] Michel F, Ferat JL (1995). Structure and activities of group II introns. Annu Rev Biochem.

[10] Lambowitz AM, Zimmerly S (2004). Mobile group II introns. Annu Rev Genet.

[11] Fedorova O, Zingler N (2007). Group II introns: structure, folding and splicing mechanism. Biol Chem.

[12] Michel F, Costa M, Westhof E (2009). The ribozyme core of group II introns: a structure in want of partners. Trends Biochem Sci.

[13] Pyle AM (2010). The tertiary structure of group II introns: implications for biological function and evolution. Crit Rev Biochem Mol Biol.

[14] Marcia M, Somarowthu S, Pyle AM (2013). Now on display: a gallery of group II intron structures at different stages of catalysis. Mob DNA.

[15] Michel F, Umesono K, Ozeki H (1989). Comparative and functional anatomy of group II catalytic introns - a review. Gene.

[16] Pyle AM, Lambowitz AM, Gesteland RF, Cech TR, Atkins JF (2006). Group II introns: ribozymes that splice RNA and invade DNA. The RNA world.

[17] Toor N, Rajashankar K, Keating KS, Pyle AM (2008). Structural basis for exon recognition by a group II intron. Nat Struct Mol Biol.

[18] Robart AR, Chan RT, Peters JK, Rajashankar KR, Toor N (2014). Crystal structure of a eukaryotic group II intron lariat. Nature.

[19] Michel F, Jacquier A (1987). Long-range intron-exon and intron-intron pairings involved in self-splicing of class II catalytic introns. Cold Spring Harb Symp Quant Biol.

[20] Jacquier A, Michel F (1990). Base-pairing interactions involving the 5′ and 3′-terminal nucleotides of group II self-splicing introns. J Mol Biol.

[21] Costa M, Michel F, Westhof E (2000). A three-dimensional perspective on exon binding by a group II self-splicing intron. EMBO J.

[22] Konforti BB, Abramovitz DL, Duarte CM, Karpeisky A, Beigelman L, Pyle AM (1998). Ribozyme catalysis from the major groove of group II intron domain 5. Mol Cell.

[23] Gordon PM, Piccirilli JA (2001). Metal ion coordination by the AGC triad in domain 5 contributes to group II intron catalysis. Nat Struct Biol.

[24] Schmelzer C, Schweyen RJ (1986). Self-splicing of group II introns *in vitro*: mapping of the branch point and mutational inhibition of lariat formation. Cell.

[25] van der Veen R, Arnberg AC, van der Horst G, Bonen L, Tabak HF, Grivell LA (1986). Excised group II introns in yeast mitochondria are lariats and can be formed by self-splicing *in vitro*. Cell.

[26] Michel F, Lang BF (1985). Mitochondrial class II introns encode proteins related to the reverse transcriptases of retroviruses. Nature.

[27] Mohr G, Perlman PS, Lambowitz AM (1993). Evolutionary relationships among group II intron-encoded proteins and identification of a conserved domain that may be related to maturase function. Nucleic Acids Res.

[28] Zimmerly S, Hausner G, Wu X (2001). Phylogenetic relationships among group II intron ORFs. Nucleic Acids Res.

[29] Blocker FJ, Mohr G, Conlan LH, Qi L, Belfort M, Lambowitz AM (2005). Domain structure and three-dimensional model of a group II intron-encoded reverse transcriptase. RNA.

[30] Gorbalenya AE (1994). Self-splicing group I and group II introns encode homologous (putative) DNA endonucleases of a new family. Protein Sci.

[31] Zimmerly S, Guo H, Perlman PS, Lambowitz AM (1995). Group II intron mobility occurs by target DNA-primed reverse transcription. Cell.

[32] San Filippo J, Lambowitz AM (2002). Characterization of the C-terminal DNA-binding/DNA endonuclease region of a group II intron-encoded protein. J Mol Biol.

[33] Jimenez-Zurdo JI, Garcia-Rodriguez FM, Barrientos-Duran A, Toro N (2003). DNA target site requirements for homing *in vivo* of a bacterial group II intron encoding a protein lacking the DNA endonuclease domain. J Mol Biol.

[34] Moran JV, Mecklenburg KL, Sass P, Belcher SM, Mahnke D, Lewin A (1994). Splicing defective mutants of the COXI gene of yeast mitochondrial DNA: initial definition of the maturase domain of the group II intron aI2. Nucleic Acids Res.

[35] Wank H, SanFilippo J, Singh RN, Matsuura M, Lambowitz AM (1999). A reverse transcriptase/maturase promotes splicing by binding at its own coding segment in a group II intron RNA. Mol Cell.

[36] Matsuura M, Noah JW, Lambowitz AM (2001). Mechanism of maturase-promoted group II intron splicing. EMBO J.

[37] Noah JW, Lambowitz AM (2003). Effects of maturase binding and Mg2+ concentration on group II intron RNA folding investigated by UV cross-linking. Biochemistry.

[38] Cui X, Matsuura M, Wang Q, Ma H, Lambowitz AM (2004). A group II intron-encoded maturase functions preferentially in cis and requires both the reverse transcriptase and X domains to promote RNA splicing. J Mol Biol.

[39] Saldanha R, Chen B, Wank H, Matsuura M, Edwards J, Lambowitz AM (1999). RNA and protein catalysis in group II intron splicing and mobility reactions using purified components. Biochemistry.

[40] Guo H, Zimmerly S, Perlman PS, Lambowitz AM (1997). Group II intron endonucleases use both RNA and protein subunits for recognition of specific sequences in double-stranded DNA. EMBO J.

[41] Cousineau B, Smith D, Lawrence-Cavanagh S, Mueller JE, Yang J, Mills D (1998). Retrohoming of a bacterial group II intron: mobility via complete reverse splicing, independent of homologous DNA recombination. Cell.

[42] Mohr G, Smith D, Belfort M, Lambowitz AM (2000). Rules for DNA target-site recognition by a lactococcal group II intron enable retargeting of the intron to specific DNA sequences. Genes Dev.

[43] Singh NN, Lambowitz AM (2001). Interaction of a group II intron ribonucleoprotein endonuclease with its DNA target site investigated by DNA footprinting and modification interference. J Mol Biol.

[44] Robart AR, Zimmerly S (2005). Group II intron retroelements: function and diversity. Cytogenet Genome Res.

[45] Matsuura M, Saldanha R, Ma H, Wank H, Yang J, Mohr G (1997). A bacterial group II intron encoding reverse transcriptase, maturase, and DNA endonuclease activities: biochemical demonstration of maturase activity and insertion of new genetic information within the intron. Genes Dev.

[46] Eskes R, Yang J, Lambowitz AM, Perlman PS (1997). Mobility of yeast mitochondrial group II introns: engineering a new site specificity and retrohoming via full reverse splicing. Cell.

[47] Smith D, Zhong J, Matsuura M, Lambowitz AM, Belfort M (2005). Recruitment of host functions suggests a repair pathway for late steps in group II intron retrohoming. Genes Dev.

[48] Contreras LM, Huang T, Piazza CL, Smith D, Qu G, Gelderman G (2013). Group II intron-ribosome association protects intron RNA from degradation. RNA.

[49] Dai L, Zimmerly S (2002). Compilation and analysis of group II intron insertions in bacterial genomes: evidence for retroelement behavior. Nucleic Acids Res.

[50] Simon DM, Clarke NA, McNeil BA, Johnson I, Pantuso D, Dai L (2008). Group II introns in eubacteria and archaea: ORF-less introns and new varieties. RNA.

[51] Candales MA, Duong A, Hood KS, Li T, Neufeld RA, Sun R (2012). Database for bacterial group II introns. Nucleic Acids Res.

[52] Granlund M, Michel F, Norgren M (2001). Mutually exclusive distribution of IS1548 and GBSi1, an active group II intron identified in human isolates of group B streptococci. J Bacteriol.

[53] Toor N, Hausner G, Zimmerly S (2001). Coevolution of group II intron RNA structures with their intron-encoded reverse transcriptases. RNA.

[54] Martinez-Abarca F, Toro N (2000). Group II introns in the bacterial world. Mol Microbiol.

[55] Toro N (2003). Bacteria and archaea group II introns: additional mobile genetic elements in the environment. Environ Microbiol.

[56] Toro N, Molina-Sanchez MD, Fernandez-Lopez M (2002). Identification and characterization of bacterial class E group II introns. Gene.

[57] Simon DM, Kelchner SA, Zimmerly S (2009). A broadscale phylogenetic analysis of group II intron RNAs and intron-encoded reverse transcriptases. Mol Biol Evol.

[58] Shub DA, Goodrich-Blair H, Eddy SR (1994). Amino acid sequence motif of group I intron endonucleases is conserved in open reading frames of group II introns. Trends Biochem Sci.

[59] Ichiyanagi K, Beauregard A, Lawrence S, Smith D, Cousineau B, Belfort M (2002). Retrotransposition of the Ll.LtrB group II intron proceeds predominantly via reverse splicing into DNA targets. Mol Microbiol.

[60] Zhong J, Lambowitz AM (2003). Group II intron mobility using nascent strands at DNA replication forks to prime reverse transcription. EMBO J.

[61] Ichiyanagi K, Beauregard A, Belfort M (2003). A bacterial group II intron favors retrotransposition into plasmid targets. Proc Natl Acad Sci U S A.

[62] Munoz-Adelantado E, San Filippo J, Martinez-Abarca F, Garcia-Rodriguez FM, Lambowitz AM, Toro N (2003). Mobility of the Sinorhizobium meliloti group II intron RmInt1 occurs by reverse splicing into DNA, but requires an unknown reverse transcriptase priming mechanism. J Mol Biol.

[63] Sunde M (2005). Class I, integron with a group II intron detected in an Escherichia coli strain from a free-range reindeer. Antimicrob Agents Chemother.

[64] Robart AR, Seo W, Zimmerly S (2007). Insertion of group II intron retroelements after intrinsic transcriptional terminators. Proc Natl Acad Sci U S A.

[65] Quiroga C, Roy PH, Centron D (2008). The S.ma.I2 class C group II intron inserts at integron attC sites. Microbiology.

[66] Quiroga C, Centron D (2009). Using genomic data to determine the diversity and distribution of target site motifs recognized by class C-attC group II introns. J Mol Evol.

[67] Leon G, Roy PH (2009). Potential role of group IIC-attC introns in integron cassette formation. J Bacteriol.

[68] Leon G, Roy PH (2009). Group IIC intron mobility into attC sites involves a bulged DNA stem-loop motif. RNA.

[69] Leon G, Quiroga C, Centron D, Roy PH (2010). Diversity and strength of internal outward-oriented promoters in group IIC-attC introns. Nucleic Acids Res.

[70] Zoschke R, Nakamura M, Liere K, Sugiura M, Borner T, Schmitz-Linneweber C (2010). An organellar maturase associates with multiple group II introns. Proc Natl Acad Sci U S A.

[71] Brown GG, Colas Des Francs-Small C, Ostersetzer-Biran O (2014). Group II intron splicing factors in plant mitochondria. Front Plant Sci.

[72] Vogel J, Hubschmann T, Borner T, Hess WR (1997). Splicing and intron-internal RNA editing of trnK-matK transcripts in barley plastids: support for MatK as an essential splice factor. J Mol Biol.

[73] Toor N, Zimmerly S (2002). Identification of a family of group II introns encoding LAGLIDADG ORFs typical of group I introns. RNA.

[74] Li CF, Costa M, Bassi G, Lai YK, Michel F (2011). Recurrent insertion of 5′-terminal nucleotides and loss of the branchpoint motif in lineages of group II introns inserted in mitochondrial preribosomal RNAs. RNA.

[75] Monteiro-Vitorello CB, Hausner G, Searles DB, Gibb EA, Fulbright DW, Bertrand H (2009). The Cryphonectria parasitica mitochondrial rns gene: plasmid-like elements, introns and homing endonucleases. Fungal Genet Biol.

[76] Salman V, Amann R, Shub DA, Schulz-Vogt HN (2012). Multiple self-splicing introns in the 16S rRNA genes of giant sulfur bacteria. Proc Natl Acad Sci U S A.

[77] Mullineux ST, Costa M, Bassi GS, Michel F, Hausner G (2010). A group II intron encodes a functional LAGLIDADG homing endonuclease and self-splices under moderate temperature and ionic conditions. RNA.

[78] Pfeifer A, Martin B, Kamper J, Basse CW (2012). The mitochondrial LSU rRNA group II intron of Ustilago maydis encodes an active homing endonuclease likely involved in intron mobility. PLoS One.

[79] Saldanha R, Mohr G, Belfort M, Lambowitz AM (1993). Group I and group II introns. FASEB J.

[80] Mullineux ST, Willows K, Hausner G (2011). Evolutionary dynamics of the mS952 intron: a novel mitochondrial group II intron encoding a LAGLIDADG homing endonuclease gene. J Mol Evol.

[81] Unseld M, Marienfeld JR, Brandt P, Brennicke A (1997). The mitochondrial genome of Arabidopsis thaliana contains 57 genes in 366,924 nucleotides. Nat Genet.

[82] Sato S, Nakamura Y, Kaneko T, Asamizu E, Tabata S (1999). Complete structure of the chloroplast genome of Arabidopsis thaliana. DNA Res.

[83] Turmel M, Otis C, Lemieux C (2003). The mitochondrial genome of Chara vulgaris: insights into the mitochondrial DNA architecture of the last common ancestor of green algae and land plants. Plant Cell.

[84] Yin LF, Hu MJ, Wang F, Kuang H, Zhang Y, Schnabel G (2012). Frequent gain and loss of introns in fungal cytochrome b genes. PLoS One.

[85] Guo W, Mower JP (2013). Evolution of plant mitochondrial intron-encoded maturases: frequent lineage-specific loss and recurrent intracellular transfer to the nucleus. J Mol Evol.

[86] Hafez M, Majer A, Sethuraman J, Rudski SM, Michel F, Hausner G (2013). The mtDNA rns gene landscape in the Ophiostomatales and other fungal taxa: twintrons, introns, and intron-encoded proteins. Fungal Genet Biol.

[87] Meng Q, Wang Y, Liu XQ (2005). An intron-encoded protein assists RNA splicing of multiple similar introns of different bacterial genes. J Biol Chem.

[88] McNeil BA, Simon DM, Zimmerly S (2014). Alternative splicing of a group II intron in a surface layer protein gene in Clostridium tetani. Nucleic Acids Res.

[89] McNeil BA, Zimmerly S (2014). Novel RNA structural features of an alternatively splicing group II intron from Clostridium tetani. RNA.

[90] Abebe M, Candales MA, Duong A, Hood KS, Li T, Neufeld RA (2013). A pipeline of programs for collecting and analyzing group II intron retroelement sequences from GenBank. Mob DNA.

[91] Carrillo C, Chapdelaine Y, Bonen L (2001). Variation in sequence and RNA editing within core domains of mitochondrial group II introns among plants. Mol Gen Genet.

[92] Li-Pook-Than J, Bonen L (2006). Multiple physical forms of excised group II intron RNAs in wheat mitochondria. Nucleic Acids Res.

[93] Barkan A, Daniell H, Chase C (2004). Intron splicing in plant organelles. Molecular biology and biotechnology of plant organelles.

[94] Germain A, Hotto AM, Barkan A, Stern DB (2013). RNA processing and decay in plastids. Wiley Interdiscip Rev RNA.

[95] Niemer I, Schmelzer C, Borner GV (1995). Overexpression of DEAD box protein pMSS116 promotes ATP-dependent splicing of a yeast group II intron *in vitro*. Nucleic Acids Res.

[96] Hammani K, Barkan A (2014). An mTERF domain protein functions in group II intron splicing in maize chloroplasts. Nucleic Acids Res.

[97] Jenkins BD, Kulhanek DJ, Barkan A (1997). Nuclear mutations that block group II RNA splicing in maize chloroplasts reveal several intron classes with distinct requirements for splicing factors. Plant Cell.

[98] Ostersetzer O, Cooke AM, Watkins KP, Barkan A (2005). CRS1, a chloroplast group II intron splicing factor, promotes intron folding through specific interactions with two intron domains. Plant Cell.

[99] Asakura Y, Barkan A (2006). Arabidopsis orthologs of maize chloroplast splicing factors promote splicing of orthologous and species-specific group II introns. Plant Physiol.

[100] Asakura Y, Bayraktar OA, Barkan A (2008). Two CRM protein subfamilies cooperate in the splicing of group IIB introns in chloroplasts. RNA.

[101] Zmudjak M, Colas Des Francs-Small C, Keren I, Shaya F, Belausov E, Small I (2013). mCSF1, a nucleus-encoded CRM protein required for the processing of many mitochondrial introns, is involved in the biogenesis of respiratory complexes I and IV in Arabidopsis. New Phytol.

[102] Mohr G, Lambowitz AM (2003). Putative proteins related to group II intron reverse transcriptase/maturases are encoded by nuclear genes in higher plants. Nucleic Acids Res.

[103] Nakagawa N, Sakurai N (2006). A mutation in At-nMat1a, which encodes a nuclear gene having high similarity to group II intron maturase, causes impaired splicing of mitochondrial NAD4 transcript and altered carbon metabolism in Arabidopsis thaliana. Plant Cell Physiol.

[104] Keren I, Bezawork-Geleta A, Kolton M, Maayan I, Belausov E, Levy M (2009). AtnMat2, a nuclear-encoded maturase required for splicing of group-II introns in Arabidopsis mitochondria. RNA.

[105] Keren I, Tal L, Colas Des Francs-Small C, Araujo WL, Shevtsov S, Shaya F (2012). nMAT1, a nuclear-encoded maturase involved in the trans-splicing of nad1 intron 1, is essential for mitochondrial complex I assembly and function. Plant J.

[106] Copertino DW, Hallick RB (1993). Group II and group III introns of twintrons: potential relationships with nuclear pre-mRNA introns. Trends Biochem Sci.

[107] Bonen L (1993). Trans-splicing of pre-mRNA in plants, animals, and protists. FASEB J.

[108] Bonen L (2008). Cis- and trans-splicing of group II introns in plant mitochondria. Mitochondrion.

[109] Knoop V, Altwasser M, Brennicke A (1997). A tripartite group II intron in mitochondria of an angiosperm plant. Mol Gen Genet.

[110] Choquet Y, Goldschmidt-Clermont M, Girard-Bascou J, Kuck U, Bennoun P, Rochaix JD (1988). Mutant phenotypes support a trans-splicing mechanism for the expression of the tripartite psaA gene in the C reinhardtii chloroplast. Cell.

[111] Goldschmidt-Clermont M, Choquet Y, Girard-Bascou J, Michel F, Schirmer-Rahire M, Rochaix JD (1991). A small chloroplast RNA may be required for trans-splicing in Chlamydomonas reinhardtii. Cell.

[112] Malek O, Knoop V (1998). Trans-splicing group II introns in plant mitochondria: the complete set of cis-arranged homologs in ferns, fern allies, and a hornwort. RNA.

[113] Qiu YL, Palmer JD (2004). Many independent origins of trans splicing of a plant mitochondrial group II intron. J Mol Evol.

[114] Stabell FB, Tourasse NJ, Ravnum S, Kolsto AB (2007). Group II intron in Bacillus cereus has an unusual 3′ extension and splices 56 nucleotides downstream of the predicted site. Nucleic Acids Res.

[115] Stabell FB, Tourasse NJ, Kolsto AB (2009). A conserved 3′ extension in unusual group II introns is important for efficient second-step splicing. Nucleic Acids Res.

[116] Tourasse NJ, Stabell FB, Kolsto AB (2010). Structural and functional evolution of group II intron ribozymes: insights from unusual elements carrying a 3′ extension. N Biotechnol.

[117] Jenkins KP, Hong L, Hallick RB (1995). Alternative splicing of the Euglena gracilis chloroplast roaA transcript. RNA.

[118] Copertino DW, Hallick RB (1991). Group II twintron: an intron within an intron in a chloroplast cytochrome b-559 gene. EMBO J.

[119] Robart AR, Montgomery NK, Smith KL, Zimmerly S (2004). Principles of 3′ splice site selection and alternative splicing for an unusual group II intron from Bacillus anthracis. RNA.

[120] Copertino DW, Christopher DA, Hallick RB (1991). A mixed group II/group III twintron in the Euglena gracilis chloroplast ribosomal protein S3 gene: evidence for intron insertion during gene evolution. Nucleic Acids Res.

[121] Hong L, Hallick RB (1994). A group III intron is formed from domains of two individual group II introns. Genes Dev.

[122] Nakamura Y, Kaneko T, Sato S, Ikeuchi M, Katoh H, Sasamoto S (2002). Complete genome structure of the thermophilic cyanobacterium Thermosynechococcus elongatus BP-1. DNA Res.

[123] Dai L, Zimmerly S (2003). ORF-less and reverse-transcriptase-encoding group II introns in archaebacteria, with a pattern of homing into related group II intron ORFs. RNA.

[124] Sheveleva EV, Hallick RB (2004). Recent horizontal intron transfer to a chloroplast genome. Nucleic Acids Res.

[125] Odom OW, Shenkenberg DL, Garcia JA, Herrin DL (2004). A horizontally acquired group II intron in the chloroplast psbA gene of a psychrophilic Chlamydomonas: *in vitro* self-splicing and genetic evidence for maturase activity. RNA.

[126] Khan H, Archibald JM (2008). Lateral transfer of introns in the cryptophyte plastid genome. Nucleic Acids Res.

[127] Kamikawa R, Masuda I, Demura M, Oyama K, Yoshimatsu S, Kawachi M (2009). Mitochondrial group II introns in the raphidophycean flagellate Chattonella spp suggest a diatom-to-Chattonella lateral group II intron transfer. Protist.

[128] Hardy CM, Clark-Walker GD (1991). Nucleotide sequence of the COX1 gene in Kluyveromyces lactis mitochondrial DNA: evidence for recent horizontal transfer of a group II intron. Curr Genet.

[129] Leclercq S, Giraud I, Cordaux R (2011). Remarkable abundance and evolution of mobile group II introns in Wolbachia bacterial endosymbionts. Mol Biol Evol.

[130] Dai L, Zimmerly S (2002). The dispersal of five group II introns among natural populations of Escherichia coli. RNA.

[131] Tourasse NJ, Kolsto AB (2008). Survey of group I and group II introns in 29 sequenced genomes of the Bacillus cereus group: insights into their spread and evolution. Nucleic Acids Res.

[132] Toro N, Martinez-Abarca F (2013). Comprehensive phylogenetic analysis of bacterial group II intron-encoded ORFs lacking the DNA endonuclease domain reveals new varieties. PLoS One.

[133] Fontaine JM, Goux D, Kloareg B, Loiseaux-de GS (1997). The reverse-transcriptase-like proteins encoded by group II introns in the mitochondrial genome of the brown alga Pylaiella littoralis belong to two different lineages which apparently coevolved with the group II ribosyme lineages. J Mol Evol.

[134] Goddard MR, Burt A (1999). Recurrent invasion and extinction of a selfish gene. Proc Natl Acad Sci U S A.

[135] Burt A, Koufopanou V (2004). Homing endonuclease genes: the rise and fall and rise again of a selfish element. Curr Opin Genet Dev.

[136] Haugen P, Simon DM, Bhattacharya D (2005). The natural history of group I introns. Trends Genet.

[137] Haugen P, Wikmark OG, Vader A, Coucheron DH, Sjottem E, Johansen SD (2005). The recent transfer of a homing endonuclease gene. Nucleic Acids Res.

[138] Adamidi C, Fedorova O, Pyle AM (2003). A group II intron inserted into a bacterial heat-shock operon shows autocatalytic activity and unusual thermostability. Biochemistry.

[139] Chee GJ, Takami H (2005). Housekeeping recA gene interrupted by group II intron in the thermophilic Geobacillus kaustophilus. Gene.

[140] Pombert JF, James ER, Janouskovec J, Keeling PJ (2012). Evidence for transitional stages in the evolution of euglenid group II introns and twintrons in the Monomorphina aenigmatica plastid genome. PLoS One.

[141] Wiegert KE, Bennett MS, Triemer RE (2013). Tracing patterns of chloroplast evolution in euglenoids: contributions from Colacium vesiculosum and Strombomonas acuminata (Euglenophyta). J Eukaryot Microbiol.

[142] Curcio MJ, Belfort M (1996). Retrohoming: cDNA-mediated mobility of group II introns requires a catalytic RNA. Cell.

[143] Simon DM, Zimmerly S (2008). A diversity of uncharacterized reverse transcriptases in bacteria. Nucleic Acids Res.

[144] Weinberg Z, Perreault J, Meyer MM, Breaker RR (2009). Exceptional structured noncoding RNAs revealed by bacterial metagenome analysis. Nature.

[145] Weinberg Z, Wang JX, Bogue J, Yang J, Corbino K, Moy RH (2010). Comparative genomics reveals 104 candidate structured RNAs from bacteria, archaea, and their metagenomes. Genome Biol.

[146] Storz G, Vogel J, Wassarman KM (2011). Regulation by small RNAs in bacteria: expanding frontiers. Mol Cell.

[147] Toor N, Keating KS, Taylor SD, Pyle AM (2008). Crystal structure of a self-spliced group II intron. Science.

[148] Rest JS, Mindell DP (2003). Retroids in archaea: phylogeny and lateral origins. Mol Biol Evol.

[149] Sharp PA (1985). On the origin of RNA splicing and introns. Cell.

[150] Cech TR (1986). The generality of self-splicing RNA: relationship to nuclear mRNA splicing. Cell.

[151] Sharp PA (1991). Five easy pieces. Science.

[152] Weiner AM (1993). mRNA splicing and autocatalytic introns: distant cousins or the products of chemical determinism?. Cell.

[153] Moore MJ, Sharp PA (1993). Evidence for two active sites in the spliceosome provided by stereochemistry of pre-mRNA splicing. Nature.

[154] Maschhoff KL, Padgett RA (1993). The stereochemical course of the first step of pre-mRNA splicing. Nucleic Acids Res.

[155] Padgett RA, Podar M, Boulanger SC, Perlman PS (1994). The stereochemical course of group II intron self-splicing. Science.

[156] Podar M, Perlman PS, Padgett RA (1998). The two steps of group II intron self-splicing are mechanistically distinguishable. RNA.

[157] Rajagopal J, Doudna JA, Szostak JW (1989). Stereochemical course of catalysis by the Tetrahymena ribozyme. Science.

[158] McSwiggen JA, Cech TR (1989). Stereochemistry of RNA cleavage by the Tetrahymena ribozyme and evidence that the chemical step is not rate-limiting. Science.

[159] Valadkhan S (2013). The role of snRNAs in spliceosomal catalysis. Prog Mol Biol Transl Sci.

[160] Chanfreau G, Jacquier A (1993). Interaction of intronic boundaries is required for the second splicing step efficiency of a group II intron. EMBO J.

[161] Parker R, Siliciano PG (1993). Evidence for an essential non-Watson-Crick interaction between the first and last nucleotides of a nuclear pre-mRNA intron. Nature.

[162] Ruis BL, Kivens WJ, Siliciano PG (1994). The interaction between the first and last intron nucleotides in the second step of pre-mRNA splicing is independent of other conserved intron nucleotides. Nucleic Acids Res.

[163] Shukla GC, Padgett RA (2002). A catalytically active group II intron domain 5 can function in the U12-dependent spliceosome. Mol Cell.

[164] Fica SM, Tuttle N, Novak T, Li NS, Lu J, Koodathingal P (2013). RNA catalyses nuclear pre-mRNA splicing. Nature.

[165] Fica SM, Mefford MA, Piccirilli JA, Staley JP (2014). Evidence for a group II intron-like catalytic triplex in the spliceosome. Nat Struct Mol Biol.

[166] Galej WP, Oubridge C, Newman AJ, Nagai K (2013). Crystal structure of Prp8 reveals active site cavity of the spliceosome. Nature.

[167] Dlakic M, Mushegian A (2011). Prp8, the pivotal protein of the spliceosomal catalytic center, evolved from a retroelement-encoded reverse transcriptase. RNA.

[168] Newman AJ, Norman C (1992). U5 snRNA interacts with exon sequences at 5′ and 3′ splice sites. Cell.

[169] Hetzer M, Wurzer G, Schweyen RJ, Mueller MW (1997). Trans-activation of group II intron splicing by nuclear U5 snRNA. Nature.

[170] O’Keefe RT, Norman C, Newman AJ (1996). The invariant U5 snRNA loop 1 sequence is dispensable for the first catalytic step of pre-mRNA splicing in yeast. Cell.

[171] Lesser CF, Guthrie C (1993). Mutations in U6 snRNA that alter splice site specificity: implications for the active site. Science.

[172] Kandels-Lewis S, Seraphin B (1993). Involvement of U6 snRNA in 5′ splice site selection. Science.

[173] Boudvillain M, de Lencastre A, Pyle AM (2000). A tertiary interaction that links active-site domains to the 5′ splice site of a group II intron. Nature.

[174] Rhode BM, Hartmuth K, Westhof E, Luhrmann R (2006). Proximity of conserved U6 and U2 snRNA elements to the 5′ splice site region in activated spliceosomes. EMBO J.

[175] Anokhina M, Bessonov S, Miao Z, Westhof E, Hartmuth K, Luhrmann R (2013). RNA structure analysis of human spliceosomes reveals a compact 3D arrangement of snRNAs at the catalytic core. EMBO J.

[176] Staley JP, Guthrie C (1998). Mechanical devices of the spliceosome: motors, clocks, springs, and things. Cell.

[177] Madhani HD, Guthrie C (1992). A novel base-pairing interaction between U2 and U6 snRNAs suggests a mechanism for the catalytic activation of the spliceosome. Cell.

[178] Reddy R, Henning D, Epstein P, Busch H (1981). Primary and secondary structure of U2 snRNA. Nucleic Acids Res.

[179] Guthrie C, Patterson B (1988). Spliceosomal snRNAs. Annu Rev Genet.

[180] Sashital DG, Cornilescu G, McManus CJ, Brow DA, Butcher SE (2004). U2-U6 RNA folding reveals a group II intron-like domain and a four-helix junction. Nat Struct Mol Biol.

[181] Dunn EA, Rader SD (2010). Secondary structure of U6 small nuclear RNA: implications for spliceosome assembly. Biochem Soc Trans.

[182] Will CL, Luhrmann R (2011). Spliceosome structure and function. Cold Spring Harb Perspect Biol.

[183] Mefford MA, Staley JP (2009). Evidence that U2/U6 helix I promotes both catalytic steps of pre-mRNA splicing and rearranges in between these steps. RNA.

[184] Irimia M, Roy SW (2014). Origin of spliceosomal introns and alternative splicing. Cold Spring Harb Perspect Biol.

[185] Andersson JO, Sjogren AM, Horner DS, Murphy CA, Dyal PL, Svard SG (2007). A genomic survey of the fish parasite Spironucleus salmonicida indicates genomic plasticity among diplomonads and significant lateral gene transfer in eukaryote genome evolution. BMC Genomics.

[186] Lane CE, van den Heuvel K, Kozera C, Curtis BA, Parsons BJ, Bowman S (2007). Nucleomorph genome of Hemiselmis andersenii reveals complete intron loss and compaction as a driver of protein structure and function. Proc Natl Acad Sci U S A.

[187] Koonin EV (2009). Intron-dominated genomes of early ancestors of eukaryotes. J Hered.

[188] Martin W, Koonin EV (2006). Introns and the origin of nucleus-cytosol compartmentalization. Nature.

[189] Belhocine K, Mak AB, Cousineau B (2007). Trans-splicing of the Ll.LtrB group II intron in Lactococcus lactis. Nucleic Acids Res.

[190] Chalamcharla VR, Curcio MJ, Belfort M (2010). Nuclear expression of a group II intron is consistent with spliceosomal intron ancestry. Genes Dev.

[191] Xiong Y, Eickbush TH (1990). Origin and evolution of retroelements based upon their reverse transcriptase sequences. EMBO J.

[192] Grainger RJ, Beggs JD (2005). Prp8 protein: at the heart of the spliceosome. RNA.

[193] Koonin EV (2006). The origin of introns and their role in eukaryogenesis: a compromise solution to the introns-early versus introns-late debate?. Biol Direct.

[194] Belhocine K, Mak AB, Cousineau B (2008). Trans-splicing versatility of the Ll.LtrB group II intron. RNA.

[195] Ritlop C, Monat C, Cousineau B (2012). Isolation and characterization of functional tripartite group II introns using a Tn5-based genetic screen. PLoS One.

[196] Qu G, Dong X, Piazza CL, Chalamcharla VR, Lutz S, Curcio MJ (2014). RNA-RNA interactions and pre-mRNA mislocalization as drivers of group II intron loss from nuclear genomes. Proc Natl Acad Sci U S A.

[197] Doolittle WF (2014). The trouble with (group II) introns. Proc Natl Acad Sci U S A.

[198] Eickbush TH, Morse SS (1994). Origin and evolutionary relationships of retroelements. Evolutionary biology of viruses.

[199] Malik HS, Burke WD, Eickbush TH (1999). The age and evolution of non-LTR retrotransposable elements. Mol Biol Evol.

[200] Luan DD, Korman MH, Jakubczak JL, Eickbush TH (1993). Reverse transcription of R2Bm RNA is primed by a nick at the chromosomal target site: a mechanism for non-LTR retrotransposition. Cell.

[201] Kojima KK, Kanehisa M (2008). Systematic survey for novel types of prokaryotic retroelements based on gene neighborhood and protein architecture. Mol Biol Evol.

[202] van der Oost J, Westra ER, Jackson RN, Wiedenheft B (2014). Unravelling the structural and mechanistic basis of CRISPR-Cas systems. Nat Rev Microbiol.

[203] Burge SW, Daub J, Eberhardt R, Tate J, Barquist L, Nawrocki EP (2013). Rfam 11.0: 10 years of RNA families. Nucleic Acids Res.

